# Spatial patterns and predictor variables vary among different types of primary producers and consumers in eelgrass (*Zostera marina*) beds

**DOI:** 10.1371/journal.pone.0201791

**Published:** 2018-08-07

**Authors:** Mizuho Namba, Masahiro Nakaoka

**Affiliations:** 1 Akkeshi Marine Station, Field Science Center for Northern Biosphere, Hokkaido University, 1 Aikappu, Akkeshi, Hokkaido, Japan; 2 Graduate School of Environmental Science, Hokkaido University, Sapporo, Hokkaido, Japan; Universita degli Studi di Urbino Carlo Bo, ITALY

## Abstract

Eelgrass (*Zostera marina*) forms extensive beds in coastal and estuarine environments and provides various ecosystem functions. The aboveground part of eelgrass provides habitats for other types of primary producers such as epiphytic microalgae and for epifaunal invertebrate grazers. Because of the different sizes, generation times and resource requirements, these different types of producers and consumers may be affected by different sets of biotic/abiotic factors over multiple spatial scales. We examined the spatial variations in three functional groups of eelgrass beds (eelgrass, epiphytic microalgae and epifaunal invertebrates) and the abiotic/biotic factors responsible for these variations in three lagoons with different environmental properties at the eastern region of Hokkaido Island, Japan. The spatial scale responsible for the variation in the biomasses of the three functional groups varied, where within-lagoon variation was important for eelgrass and epifauna but among-lagoon variation was important for microalgae. The environmental predictors for the observed spatial variations also differed among the different functional groups, with variation in eelgrass biomass related to depth, nutrient and salinity, epiphytes to water temperature, eelgrass biomass and water column chlorophyll and epifauna mainly to eelgrass biomass. These results revealed that the level of importance of among- and within-lagoon environmental gradients vary in the different functional groups of the eelgrass bed community. The large-scale variation in pelagic productivity, which is tightly related to the ocean current regimes, is likely responsible for the great among-lagoon variation in microalgae. The local variations in environmental factors such as salinity and nutrients, which change with alterations in terrestrial river inputs, are likely related to the great variations in eelgrass and epifauna within the ecosystem. The observed relationship of epifauna with eelgrass biomass indicates the importance of non-trophic plant-animal interactions because epifauna utilize eelgrass as habitat. We therefore emphasize the importance of evaluating spatial variations at multiple scales to further understand the functions of coastal and estuarine ecosystems.

## Introduction

Intertidal and subtidal meadows formed by submerged aquatic vegetation (SAV) are among the most common habitat types in coastal marine ecosystems [1, 2], and these ecosystems are often compared to terrestrial forests and grasslands in terms of their ecosystem functions and services [3]. Seagrass beds are one of these ecosystems and are found in shallow coastal marine and estuarine environments around the world [4]. The ecosystem functions provided by seagrass beds include the provisioning of habitats for diverse fauna and flora through the addition of physical structures to the seafloor [2, 5] and the support of high primary and secondary productivity [6], which makes them one of the most productive ecosystems in the world [7]. Primary producers in seagrass beds consist of various plant functional groups that are separated in terms of their turnover rates and light and nutrient requirements and include groups such as seagrasses, epiphytic microalgae on seagrass blades, benthic algae and phytoplankton [8, 9]. The amount of production by various primary producers differs spatially and seasonally [10, 11]. The amount of production by epiphytic and benthic algae sometimes exceeds the production by seagrass [8]. Consumers in seagrass beds are also diverse and consist of small invertebrates such as gastropods, amphipods, shrimps, and annelids, and some vertebrates such as rabbitfish, green sea turtles, manatees and waterfowl [12, 13]. The invertebrates can be categorized as epifauna or infauna depending on where they inhabit the seagrass beds, and they utilize the food sources and habitats provided by the primary producers, as most of the invertebrates are grazers and detritivores [14].

There have been many studies that have examined the variations in primary producers and consumers in seagrass beds [15, 16]. Investigations have been conducted on plant-plant interactions, such as the competition for light and nutrients between seagrass and algae [17], and plant-animal interactions, such as the control of plant abundance by herbivores [12, 18]. For epifaunal consumers such as invertebrate herbivores, the bottom-up effects from plants on animals have been investigated, and these investigations have focused on the relationships among the abundances of associated animals, microalgae and seagrass (e.g., [19–21]). The investigations of the top-down effects have focused on the predation pressures on herbivores by predators such as fish [22]. Such plant-plant and plant-animal interactions are affected by the variations in multiple environmental factors such as temperature, salinity and nutrients [23, 24].

The different environmental factors that affect the ecosystem functions of coastal habitats, including seagrass beds, operate at different spatial scales. The abiotic factors of ocean currents and associated oceanographic factors (such as water temperatures and nutrient concentrations) affect the community structures of primary producers and consumers over large spatial scales; e.g., 100 km scale [25, 26]. At the meso scale (e.g., 10 km scale), the presence of temporal and spatial salinity gradients within lagoonal and estuarine ecosystems is common due to the changes in the tidal effects from the ocean and freshwater inputs from rivers [27]. At this spatial scale, the differences in land-use along the watersheds of the rivers that flow into lagoons and estuaries also cause variations in the amounts of nutrient input [28], while variations in physical properties, such as water depth, salinity and hydrodynamic conditions, impact the community structures and the amount of production [29–31]. At fine spatial scales (< 1 km, for example), it is commonly observed that microhabitat heterogeneity and species interactions affect the diversity and abundance of different components of organisms in a community, as mentioned above. Different functional groups of organisms likely respond differently to these environmental variables due to differences in functional traits. Microalgae within seagrass beds have much faster turnover rates than seagrasses [32] and may have different relationships with nutrient concentrations and other environmental requirements, leading to different spatial variation patterns across multiple scales compared to seagrass. Therefore, a simultaneous investigation of different types of primary producers and consumers over multiple spatial scales is necessary to understand how these functional groups interact and structure the highly productive ecosystem. Additionally, how various environmental factors in seagrass beds affect these groups of organisms should be analyzed by comparing multiple seagrass bed ecosystems that are stretched over a large spatial scale, that are different in environmental properties at meso scales, and that environmental heterogeneity is present at fine scales for more precise evaluation of ecosystem functions.

This study aims to investigate the patterns in the spatial variation of each of the major functional groups of primary producers (eelgrass *Zostera marina* and epiphytic microalgae) and consumers (invertebrate epifauna) in eelgrass beds, the most widely distributed seagrass beds in the temperate northern hemisphere [15], and to determine how they are shaped by various environmental factors. First, we determine the most prominent spatial scale of the variability in the biomass of the two groups of primary producers and epifauna by comparing the among-lagoon (20–200 km; large scale) and within-lagoon (< 20 km; meso and fine scale, but hereafter we refer to this as fine scale) variation. We then explore the abiotic and biotic factors that are related to the observed variabilities in the biomasses of different functional groups. We study three lagoons on the eastern part of Hokkaido Island, Japan (hereafter eastern Hokkaido) which exhibit differences in the effect of ocean currents and the amount of river inputs. In these lagoons, eelgrass forms extensive beds and supports high primary and secondary production, which benefits the local communities through commercial fishing of seagrass-associated secondary producers such as *Pandalus* shrimp [33–35]. Here, we hypothesized that the among-lagoon variation would be responsible for the variation in biomass if the organisms are affected more by large scale environmental factors such as the difference in water temperature, whereas within-lagoon variation would be more important if the organisms are affected by the factors associated with finer-scale nutrient or salinity gradients caused by freshwater and terrestrial inputs with river discharges.

## Materials and methods

### Study area

#### Eastern Hokkaido

**Fig 1:**
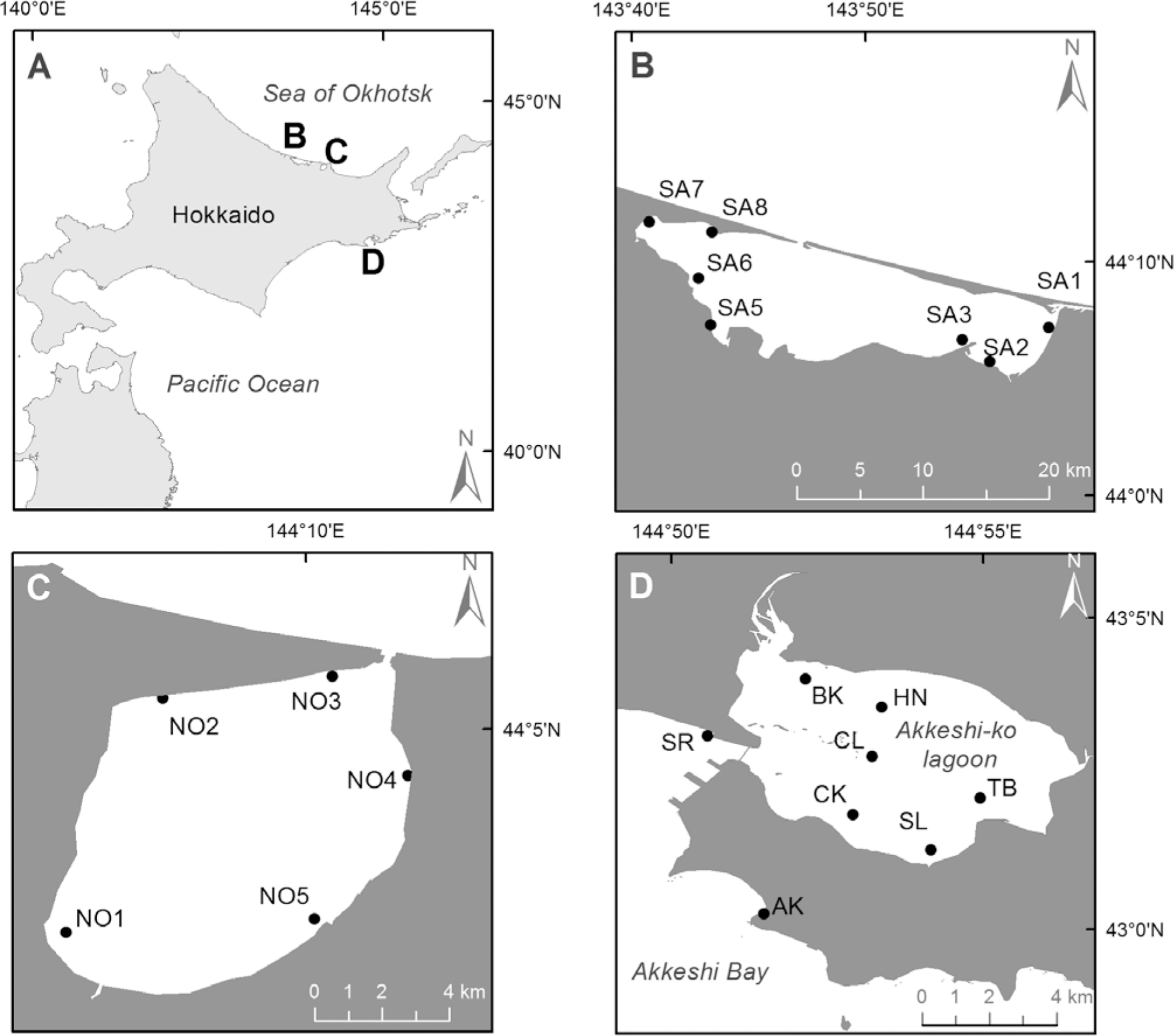
Study sites. (A) In eastern Hokkaido at the (B) Saroma-ko Lagoon, (C) Notoro-ko Lagoon, and (D) Akkeshi-ko Lagoon and Akkeshi Bay.

Eastern Hokkaido faces two different oceans: the Pacific on the southeastern side and the Sea of Okhotsk on the northeastern side (Fig 1). The coastal areas are characterized by the presence of multiple semi-enclosed estuaries and lagoons with saline to brackish water. The Oyashio cold current flows in the southwest direction along the Kuril Islands and Hokkaido coast [36], strongly affecting the Pacific Ocean side of eastern Hokkaido. The seawater in the Oyashio-influenced area is characterized by cold temperatures reaching < 0°C in the winter [36] and a high concentration of chlorophyll *a* (hereafter Chl-*a*) in the water column during the spring bloom from February to April ([36, 37], personal observation T. Isada). In the coastal areas, an autumn bloom from August to October has also been observed (personal communication T. Isada). The water mass of the current is rich in NO_3_ in the winter but is depleted in the spring when phytoplankton blooms [37].

The Soya warm current flows in the Sea of Okhotsk towards the southeast along eastern Hokkaido [38, 39]. The current dominates in May-October and is responsible for the higher salinities and temperatures than in the Oyashio current [39]. In November-April, the effects of the Soya current in the Sea of Okhotsk are weakened, and the East Sakhalin cold current becomes more influential and brings water masses with low salinities and low temperatures [40]. The timing of the spring bloom and nutrient depletion is similar to that in the Oyashio-influenced region, but the coastal areas of the Sea of Okhotsk have lower phytoplankton abundances and nutrient concentrations compared to the Pacific coastal areas [36, 37, 39]. Moreover, seasonal sea ice usually covers the Okhotsk coast from January to April [41].

For this research, no permit was required for the field sampling as the method used for the study (use of mesh bags) was exempt from the list of fishing gears that need to be declared and used with fishing permits. Moreover, the sampled plants and invertebrates did not include the commercial species or protected species.

#### Akkeshi

The Akkeshi-ko Lagoon and Akkeshi Bay are located along the Pacific side of eastern Hokkaido (Table 1; Fig 1). They are connected to each other by a narrow channel that is approximately 500 m wide and 10 m deep. The surface areas of the Akkeshi-ko Lagoon and Akkeshi Bay are 32 km^2^ and 110 km^2^, respectively. The southern part of the bay is open to the Pacific Ocean. There are three major rivers (Bekanbeushi, Tokitai, and Tobai) that flow into the Akkeshi-ko Lagoon; 98.8% of the total input to the lagoon is from the Bekanbeushi River [24], and its average daily input accounts for 5.8% of the total water volume of the lagoon [42]. The watershed of the Bekanbeushi River is mostly covered by wetlands, agricultural lands, and forests. The combination of saline water from Akkeshi Bay that is influenced by the Oyashio current and the freshwater input from the rivers makes the water mass in the Akkeshi-ko Lagoon brackish and is responsible for the various physical and chemical gradients, including the presence of temporal and spatial salinity and nutrient gradients [24, 42].

**Table 1:** Environmental conditions in the summer (June to July) and fall (August to September) 2016 at the study sites in Akkeshi, Saroma and Notoro.

Site	Water temperature (°C)		Salinity		Water Depth (m)	
	Summer	Fall	Summer	Fall	Summer	Fall
**Akkeshi**						
AK	11.8	21.1	30.9	27.0	0.2	0.8
BK	16.1	22.1	18.4	11.0	0.7	0.6
CK	12.8	21.5	21.7	23.5	1.0	1.2
CL	10.6	21.5	27.4	20.6	1.0	0.8
HN	14.1	23.0	12.3	12.0	0.9	0.7
SL	13.7	22.8	16.1	18.6	0.5	0.8
SR	10.4	20.0	23.4	19.2	0.8	1.2
TB	13.7	22.7	13.7	14.8	0.9	0.8
Mean±SD	12.9 ± 1.9	21.8 ± 1.0	20.5 ± 6.6	18.3 ± 5.5	0.8 ± 0.2	0.9 ± 0.2
**Saroma**						
SA1	18.7	17.6	32.2	31.8	1.5	2.0
SA2	n.d.	17.8	n.d.	31.6	n.d.	1.0
SA3	20.6	17.2	32.3	31.9	1.0	0.5
SA5	18.5	n.d.	32.8	n.d.	2.7	n.d.
SA6	19.7	18.2	32.3	31.3	1.1	0.8
SA7	18.1	n.d.	32.9	n.d.	1.6	n.d.
SA8	17.5	17.4	33.1	32.2	1.3	0.9
Mean±SD	18.8 ± 1.1	17.7 ± 0.4	32.6 ± 0.4	31.8 ± 0.4	1.4 ± 0.6	1.0 ± 0.5
**Notoro**						
NO1	18.5	17.2	32.7	32.5	0.7	0.4
NO2	19.3	17.7	32.9	32.3	0.9	0.6
NO3	17.0	18.1	33.1	32.2	0.7	0.8
NO4	18.5	19.3	33.1	31.6	0.4	1.3
NO5	19.1	17.7	32.5	32.6	0.4	0.6
Mean±SD	18.5 ± 0.9	18.0 ± 0.8	32.9 ± 0.3	32.2 ± 0.4	0.6 ± 0.2	0.7 ± 0.4

n.d. indicates no data available. The water depth is calculated based on the mean lower low water.

The water depth of the Akkeshi-ko Lagoon ranges between 0.8 and 1.7 m, and its floor is muddy and covered by mostly eelgrass (*Zostera marina*) except in the intertidal areas where Manila clam (*Ruditapes philippinarum*) aquaculture grounds are present [24, 43, 44]. Manila clam and Pacific oyster (*Crassostrea gigas*) farming are the main aquaculture activities in the lagoon [45]. In Akkeshi Bay, *Z*. *marina* occurs in the intertidal to shallow subtidal zones where the depth is less than 2 m, and *Z*. *asiatica* dominates in the deeper subtidal zone where the depth is limited to 5 m below the mean low water [46].

A total of 6 study sites were established on the shallow subtidal bottom of Akkeshi-ko Lagoon (BK: at the Bekanbeushi River mouth; CK: Chikarakotan; CL: Central Lagoon; HN: Horonitai; SL: South Lagoon; TB: Toubai), and 2 sites were established in Akkeshi Bay (AK: Aininkappu; SR: Shinryu) (Table 1; Fig 1). See [44] for the detailed explanation of each site and Table 1 for the physical parameters.

#### Saroma

The Saroma-ko Lagoon is located on the northeastern part of Hokkaido and is connected to the Sea of Okhotsk by one channel in the east and another channel in the west (Table 1; Fig 1). The area of the lagoon is 152 km^2^ and has an average depth of 14 m [30]. There are 8 major rivers that flow into the lagoon, with seasonal differences in the freshwater discharges, and the Saromabetsu River in the eastern part of the lagoon is the largest in terms of catchment size [47]. The watersheds of these rivers mainly consist of forests and agricultural lands. Like the Akkeshi-ko Lagoon, the mixture of seawater and freshwater, as well as the presence of water currents within the lagoon, creates a complex physical environment [48], yet the nutrient and salinity gradients within the lagoon are not as notable as the ones in Akkeshi-ko lagoon.

Eelgrass beds are found in the shallow subtidal areas near the coast of the Saroma-ko Lagoon, together with other seagrass species, *Z*. *caspitosa* and *Z*. *japonica* ([30, 49], personal observation M. Namba). The bottom of the lagoon is mainly sand. Scallop (*Mizuhopecten yessoensis*) and oyster (*Crassostrea gigas*) farming are the major primary industries in the lagoon. However, hypoxic events due to the excessive inputs of organic matter from the scallop farming sites and the rivers have been occurring in recent years [48] and are considered to be responsible for the decline of the eelgrass beds over the past decade [30].

A total of 7 study sites (SA1, 2, 3, 5, 6, 7, and 8) were established in the subtidal part of the Saroma-ko Lagoon (Table 1; Fig 1), and these sites corresponded to the periodical monitoring sites by the Hokkaido Aquaculture Promotion Corporation [50].

#### Notoro

The Notoro-ko Lagoon is located in the south of Saroma-ko lagoon on the Sea of Okhotsk (Fig 1) and has a maximum depth of 20 m [51] and an area of 58.4 km^2^ [52]. It is connected to the ocean through a channel that is 324 m side and 13 m deep, and the water exchange rate between the lagoon and the ocean is influenced by tides [51]. Although there are 11 rivers that run into the lagoon, the outfall from Ubaranai River, which is the largest, constitutes only 0.01% of the total water volume of the lagoon due to the small catchment area [52]. Thus, the effects of the rivers are much smaller than that of the ocean, and this makes both salinity and nutrient concentrations almost uniform within the lagoon (personal communication S. Chiba). The salinity of the lagoon is approximately 33, which is similar to the salinity of the Sea of Okhotsk [53]. The watershed of the Notoro-ko Lagoon is smaller than those of the Akkeshi-ko and Saroma-ko Lagoons, and the environment surrounding the lagoon is mainly agricultural lands, with some forests present on the northeastern shore.

In the Notoro-ko Lagoon, two species of seagrasses, *Z*. *marina* and *Z*. *caespitosa*, form beds on the sandy bottoms of the shallow subtidal areas [21], and *Z*. *japonica* occurs in the intertidal areas. The eelgrass beds provide habitat for the shrimp *Pandalus latirostris*, which is the main fishery resource in the lagoon [21].

A total of 5 study sites were established in the shallow subtidal part of the lagoon (NO1, 2, 3, 4, 5, Table 1; Fig 1). A site was not established in the middle of the lagoon as it is deeper than the depth limit of seagrasses.

### Field sampling

In the three lagoons, the first set of field sampling was carried out between June 23 and July 13, 2016 (hereafter ‘summer’) when the eelgrass beds are most productive in this region [11, 54]. The second set of field sampling was undertaken between August 16 and September 28, 2016 (hereafter ‘fall’). Throughout the study, the data obtained from these two seasons were analyzed separately as temporal replicates to assess the reproducibility of the spatial variation. The sampling was conducted during the daytime neap tide so that the effects of tidal currents were minimized. In the summer, SA2 in the Saroma-ko Lagoon was not accessible due to the excess amount of freshwater discharge and subsequent turbidity. In the fall, samples were not collected at SA5 and SA7 in the Saroma-ko Lagoon because the eelgrass at these sites had been decimated by a storm. The water depth in Saroma was deeper than in the other two lagoons, and the depth at SA5 was approximately 3 m. Because of the lack of eelgrass beds in the fall at SA5 where the depth was the deepest, the differences among the average depths in Saroma, Notoro, and Akkeshi were smaller in the fall.

We used memory sensors (AAQ-175 RINKO: JFE Advantech Co. Ltd., Japan, and RINKO-Profiler ASTD102-ALC-R02: JFE Advantech Co., Ltd., Japan) to measure the water temperature, salinity, and water depth at each site. For each site, water samples were collected using a plastic bucket that was washed three times prior to sampling. The water used for the Chl*-a* measurements was collected using a 138.5 ml darkened polyethylene bottle (one sample per site). For nutrient analysis, 200 ml opaque polyethylene bottles were used to collect water (one sample per site). All the bottles containing water samples were brought back to the laboratory in a darkened cooler box filled with ice.

At each site, we haphazardly collected five replicate samples of mobile invertebrates (hereafter ‘epifauna’) from the aboveground parts of eelgrass using a mesh bag (20 cm diameter, mesh size 0.1 mm) in a circular 0.0314 m^2^ area in the middle of the eelgrass beds [44]. From these samples, we obtained data on the biomass for both epifauna and eelgrass and then extrapolated the values for 1m^2^ area from the data. Microalgal samples were obtained by collecting five replicate samples of eelgrass shoots per site, and each sample was placed in a separate plastic zip bag [44] and stored in a darkened cooler box until analysis in the laboratory.

### Laboratory procedures

The water sample used for nutrient analysis was filtered through a 0.45 μm nylon membrane filter (Millex—HN Filter Unit, Merck Millipore Ltd., Tullagreen, Carrigtwohill, Ireland) into glass vials and frozen at—20 °C until analysis. The nutrient concentrations, including the amounts of total nitrogen (TN, the sum of NO_2_-N, NO_3_-N, and NH_4_-N) and PO_4_-P in the water filtrates, were measured simultaneously using an AutoAnalyzer (QuAAtro 39, BL tec, Osaka, Japan).

The non-acidification method of Welschmeyer [55] was used to analyze the Chl*-a* concentrations. For the water column Chl*-a* analysis, each water sample was filtered through GF/F glass-fiber filters (Whatman International Ltd., Maidstone, UK). To determine the abundance of epiphytic microalgae from the Chl*-a* concentration, microalgae on each eelgrass sample was scraped off using a glass slide and filtered through the GF/F filters. The filters were then extracted in 6 ml N,N-dimethylformamide for more than one day and stored at—20 °C in the dark until analysis [56]. The Chl*-a* concentration within each extract was then measured by using a fluorometer (10-AU-005-CE Fluorometer, Turner Designs, Sunnyvale, CA, USA).

The epifaunal invertebrates collected in the mesh bags were scraped from the eelgrass and filtered through a 0.5 mm sieve. The epifaunal samples retained on the sieve were then fixed with 70% ethanol, and only those with the sizes between 0.5 mm and 8 mm were counted, identified to the lowest possible taxonomic group with a dissecting microscope, and grouped into different size groups for the estimation of the ash-free dry weight using the formulas provided by Edgar [57]. In total, 19 groups of epifauna were identified to the order, class, or phylum level based on the taxonomic knowledge and information available. All eelgrass shoots were then put in small aluminum foil bags and dried at 60 °C for 48 hr or until completely dried in an oven, and they were weighed to obtain the dry mass.

### Statistical analysis

The statistical program R [58] was used for all statistical analyses (see S1 Dataset for the all data used for the analyses and S1 Text for the R commands). To assess the spatial variation in the biomasses of the different functional groups in the eelgrass bed community and determine the effects of different spatial scales, a one-factor nested analysis of variance with the sites nested in the lagoons was performed for each season and for each of the following variables; eelgrass (g dry weight per unit area: g DW m^-2^), microalgae (g Chl*-a* per unit area: g Chl*-a* m^-2^) and epifauna (g ash-free dry weight per unit area: g AFDW m^-2^). All variables were log-transformed to meet the assumptions of normality and homogeneity. The test was followed by partitioning the variance components using *VCAinference* code in the VCA package [59].

To determine the abiotic and biotic factors responsible for the variation in the three variables, linear mixed models (LMMs) with Gaussian distributions were used [60]. For the fixed factors, a list of abiotic and biotic candidate variables (water temperature, PO_4_-P, TN, depth, salinity, microalgae Chl-*a*, water Chl-*a*, eelgrass biomass) that could affect the eelgrass biomass, abundance of microalgae and epifaunal biomass was made based on previous observations and the literature (e.g. [24, 44]). Collinearity among the variables was then checked by calculating Pearson’s correlation coefficients for all pairs. Any variables that had absolute values of the coefficient greater than 0.7 [61] were removed from the list, which resulted in a list of five explanatory variables (TN, depth, water temperature, salinity, and water Chl*-a*) used for the assessment of eelgrass biomass. Microalgae Chl-*a* and PO_4_-P were removed from the list due to observed collinearity between salinity and TN, respectively. Different variable combinations were used for the microalgae biomass (TN, depth, water temperature, and water Chl-*a*, eelgrass biomass) and the epifaunal biomass (TN, depth, water temperature, water Chl*-a*, eelgrass biomass, microalgae Chl*-a*) after checking for collinearity among the variables by the abovementioned method. LMM analyses for each of the three functional groups were first carried out to examine all lagoons together (with three random factors: sites nested in the lagoon, lagoon, and season) and then for each lagoon separately (with two random factors: site and season).

The LMMs were fit with the *lmer* function in the lme4 package [62], and their p-values were obtained by using the lmerTest package [63]. The best model for each of the three producers was chosen based on the AICc, which is Akaike’s information criterion (AIC) adjusted for small sample sizes, where AIC and AICc will be equal at large sample sizes [64]. The AICc was based on restricted maximum likelihood (REML), as the selection was made among the models with nested random factors [65]. To determine the components of the variances explained (R^2^) by the fixed factors (marginal R^2^) and the combination of the fixed and random factors (conditional R^2^) [66], the *r*.*squaredGLMM* function in the MuMIn package [67] was used. The variables chosen by the best models were then plotted with linear regression lines shown only if there was a significant relationship between the two variables (linear models, *P* ≤ 0.05).

## Results

### Environmental variables

We expected to see the differences in environmental gradients among the lagoons due to water currents at larger spatial scales and the amount of river discharges at finer spatial scales. The results showed that the difference in water temperature was observed among the lagoons, and that variations in salinity and nutrient concentration were most notable within Akkeshi where the amount of river discharge was largest among the three lagoons.

**Fig 2:**
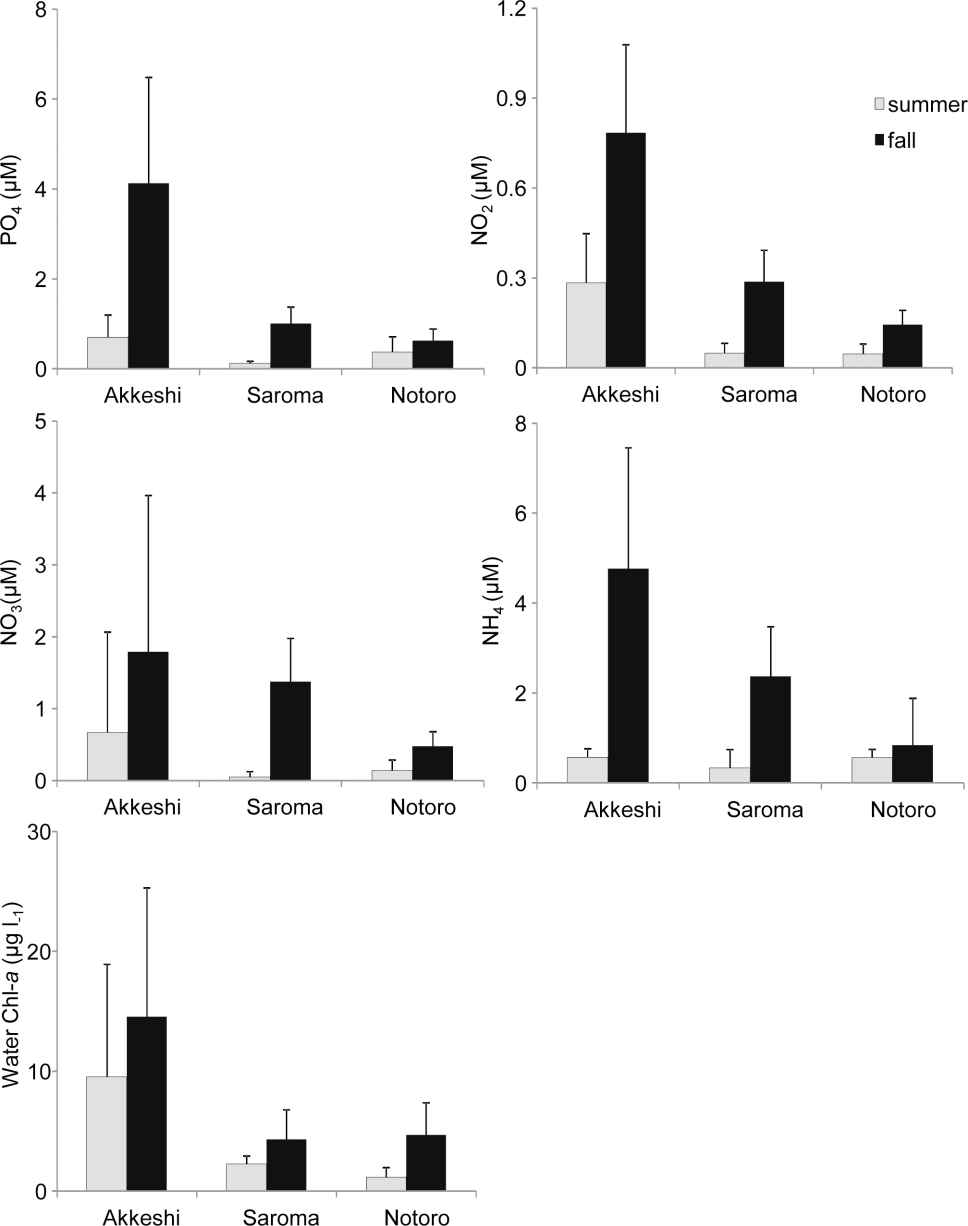
Mean (+ SD) concentrations of water nutrients and Chl*-a* concentrations in the water column. Nutrients include PO_4_, NO_2_, NO_3_, and NH_4_. n = 8 for Akkeshi in the summer and fall, n = 6 for Saroma in the summer, n = 5 for Saroma in the fall, n = 5 for Notoro in the summer and fall.

The water temperature in Akkeshi was lower overall than in Saroma and Notoro in the summer, but it was warmer than in the two lagoons on the Okhotsk coast in the fall (Table 1). The average salinity was lower in Akkeshi than in Saroma and Notoro. The salinity varied from 12.3 to 30.9 among the sites in Akkeshi, but it was homogeneous among the sites in Saroma and Notoro (Table 1). Four components of the nutrient concentrations varied among ecosystems and among sites within each ecosystem, and the water nutrient concentrations were higher in Akkeshi than Saroma and Notoro (Fig 2). In addition, the measured concentrations were more heterogeneous among the sites in Akkeshi than in Saroma or Notoro (S1 Table). The water-column Chl*-a* concentration was 2 to 3-fold higher in Akkeshi than in the two lagoons on the Okhotsk coast during both sampling times (Fig 2), and the concentrations in Saroma and Notoro were relatively homogeneous compared to those in Akkeshi.

### Eelgrass and microalgae

**Fig 3:**
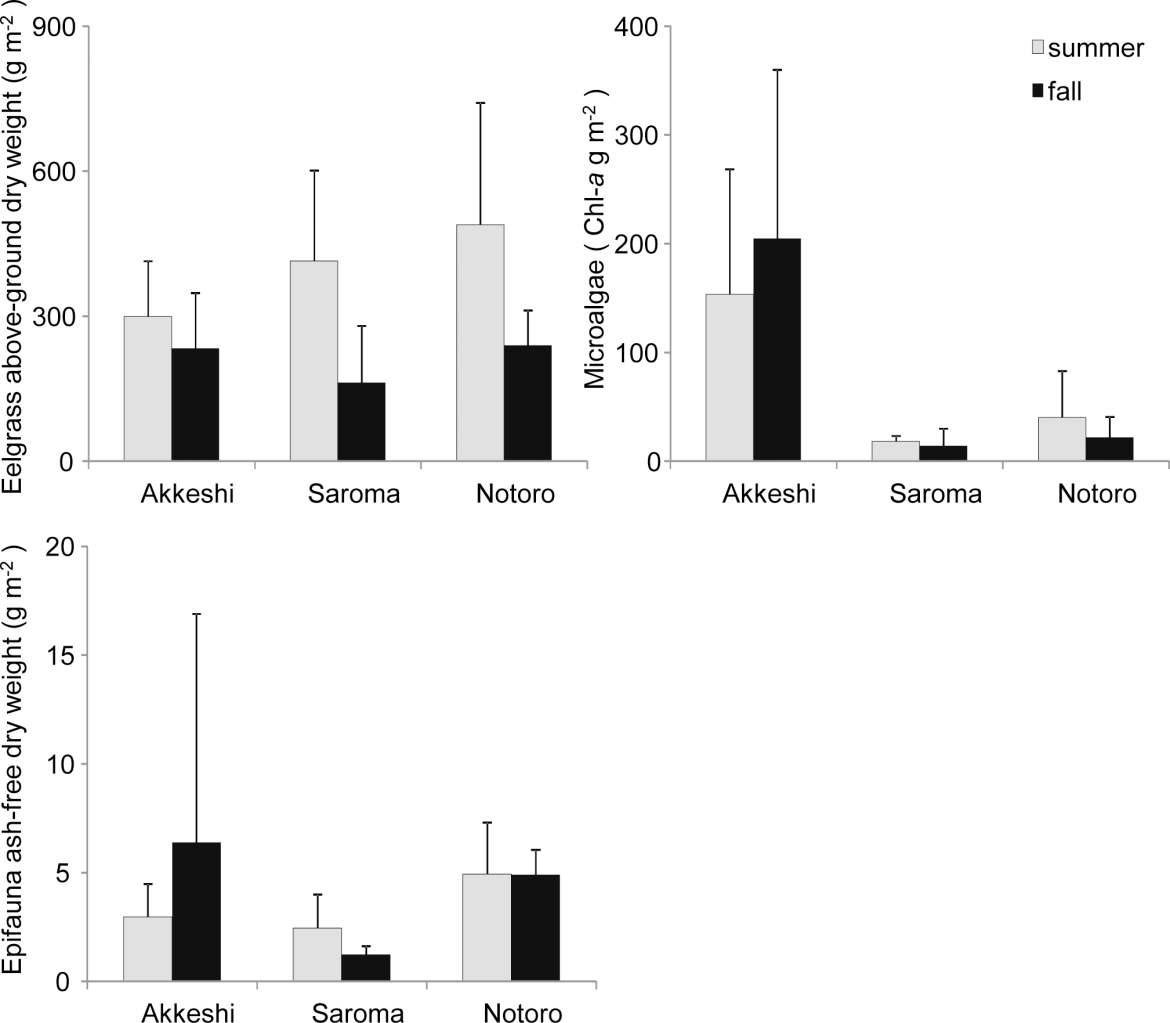
Mean (+ SD) biomass of eelgrass, microalgae, and epifauna. n = 8 for Akkeshi in the summer and fall, n = 6 for Saroma in the summer, n = 5 for Saroma in the fall, n = 5 for Notoro in the summer and fall of the above-ground dry weight of eelgrass (g m^-2^), microalgae weight (g Chl*-a* m^-2^), and ash-free dry weight of epifauna (g m^-2^) at Akkeshi, Saroma, and Notoro for the two temporal replicates (summer and fall). The error bars show the standard deviations.

The above-ground dry weight of eelgrass differed among the lagoons (two-way nested ANOVA: summer *F*_2, 74_ = 9.49, *P* < 0.001; fall *F*_2, 72_ = 11.16, *P* < 0.001) and among the sites within the lagoons (summer *F*_16, 74_ = 6.38, *P* < 0.001; fall *F*_15, 72_ = 4.88, *P* < 0.001) in both the summer and fall (Table 2; Fig 3). The partitioning of the variance components for the nested ANOVA results showed that 50 and 39% of the variances were attributed to the within-lagoon differences, 45 and 50% were attributed to the within-site differences, and only 5 and 10% of the variances were attributed to the among-lagoon differences in the summer and fall, respectively (Table 3).

**Table 2:** Mean above-ground dry weight (n = 5) of eelgrass, microalgae, and epifauna.

Site	Eelgrass		Microalgae		Epifauna	
	Summer	Fall	Summer	Fall	Summer	Fall
**Akkeshi**						
AK	172.4	194.5	21.8	23.0	1.1	1.9
BK	142.5	283.3	47.4	384.0	2.4	31.8
CK	365.6	276.4	252.9	186.8	3.7	3.7
CL	280.2	465.4	61.0	240.8	5.9	7.7
HN	440.2	98.0	135.1	109.8	2.6	1.1
SL	425.6	221.6	108.9	90.0	1.8	1.9
SR	211.5	206.2	321.8	475.6	4.1	2.2
TB	355.3	115.2	276.7	128.1	2.2	0.7
Mean±SD	295.9 ± 157.4	232.6 ± 156.5	147.3 ± 120.0	204.8 ± 161.7	3.0 ± 2.1	6.4 ± 10.6
**Saroma**						
SA1	292.8	344.7	9.3	41.5	1.4	1.4
SA2	n.d.	209.3	n.d.	12.8	n.d.	1.2
SA3	352.2	122.9	20.7	4.5	2.6	1.4
SA5	670.0	n.d.	22.7	n.d.	0.7	n.d.
SA6	506.2	68.9	17.2	2.4	4.8	0.6
SA7	145.3	n.d.	23.3	n.d.	3.6	n.d.
SA8	516.9	64.4	15.3	8.6	1.6	1.6
Mean±SD	413.9 ± 258.8	162.0 ± 129.8	18.1 ± 13.6	14.0 ± 17.0	2.4 ± 2.0	1.2 ± 0.7
**Notoro**						
NO1	608.2	138.9	51.4	16.1	5.2	3.7
NO2	816.1	228.9	92.1	32.5	7.3	5.0
NO3	548.4	243.1	15.3	8.9	7.1	6.5
NO4	260.3	239.7	9.0	32.4	2.3	5.5
NO5	210.3	344.3	32.6	17.6	2.7	3.9
Mean±SD	488.7 ± 280.6	239.0 ± 99.6	40.1 ± 42.6	21.7 ± 19.0	4.9 ± 3.5	4.9 ± 2.3

The results for eelgrass (Eelgrass, g m^-2^), microalgae abundance (Microalgae, Chl-*a* g m^-2^), and ash-free dry weight of epifauna (Epifauna, g m^-2^) in the summer and fall 2016 at the study sites in Akkeshi, Saroma, and Notoro are shown. n.d. indicates no data available.

**Table 3:** Variance components (in %) assigned to the different levels by the nested ANOVA.

	Summer					Fall				
Source	df	MS	*F*	*P*	%	df	MS	*F*	*P*	%
**Eelgrass**										
Lagoon	2	1.78	9.49	> 0.001	4.55	2	3.48	11.16	> 0.001	10.83
Lagoon:Site	16	1.20	6.38	> 0.001	50.03	15	1.52	4.88	> 0.001	38.93
Error	74	0.19			45.43	72	0.31			50.24
**Microalgae**										
Lagoon	2	35.01	80.62	> 0.001	49.81	2	75.59	233.74	> 0.001	70.01
Lagoon:Site	16	3.41	7.84	> 0.001	29.28	15	4.00	12.36	> 0.001	20.89
Error	74	0.43			20.92	71	0.32			9.10
**Epifauna**										
Lagoon	2	5.60	13.79	> 0.001	13.60	2	13.86	35.02	> 0.001	22.18
Lagoon:Site	16	2.03	5.00	> 0.001	38.38	15	4.22	10.66	> 0.001	51.27
Error	76	0.41			48.02	72	0.40			26.55

The effects of the lagoons and the sites nested in the lagoons for eelgrass, microalgae, and epifauna for two temporal replicates (summer and fall) are shown.

The amount of microalgae biomass differed significantly among the lagoons (two-way nested ANOVA: summer *F*_2, 74_ = 80.62, *P* < 0.001; fall *F*_2, 71_ = 233.74, *P* < 0.001), showing that the abundance of microalgae was much higher in Akkeshi (> 100-fold) than in the two Okhotsk lagoons (Table 2; Fig 3). The differences among the sites (two-way nested ANOVA: summer *F*_16, 74_ = 7.84, *P* < 0.001; fall *F*_15, 71_ = 12.36, *P* < 0.001) were also prominent, and the overall variations were higher in Akkeshi than in the other two lagoons. In contrast to the results for eelgrass, the partitioning of the variance components indicated that 50 and 70% of the variances were attributed to the among-lagoon differences, 29 and 21% of the variances were attributed to the within-lagoon differences, and 21 and 9% of the variances were attributed to the within-site differences in the summer and fall, respectively (Table 3).

### Epifauna

A total of 104 taxa belonging to 19 taxonomic groups were collected in this study. Gammaridea were most common in the summer in Akkeshi, followed by gastropoda. In the fall, gastropoda was dominant in most sites in Akkeshi. In the summer in Saroma, caprellidea, bivalva, and gastropoda were abundant, whereas gastropoda was the dominant group in the fall. In Notoro, gastropoda was the group that contributed most to the total biomass both in the summer and fall. See S1 Fig for the taxonomic composition of the epifauna for each lagoon and season.

The epifaunal biomass significantly differed among the lagoons (two-way nested ANOVA: summer *F*_2, 76_ = 13.79, *P* < 0.001; fall *F*_2, 72_ = 35.02, *P* < 0.001) for both seasons (Table 2; Fig 3). The lowest epifaunal biomass was observed at the sites of Saroma. There were also differences among the sites within the lagoons (two-way nested ANOVA: summer *F*_16, 76_ = 5.00, *P* < 0.001; fall *F*_15, 72_ = 10.66, *P* < 0.001). BK in Akkeshi had almost 30 times higher epifaunal biomass than the rest of the sites in Akkeshi and all sites in Notoro and Saroma. The partitioning of the variance components showed that 14 and 22% of the variances were from the among-lagoon variations, 38 and 51% were from the within-lagoon variations, and 48 and 27% were from the within-site variations in the summer and fall, respectively (Table 3).

### Factors responsible for the spatial variation

**Fig 4:**
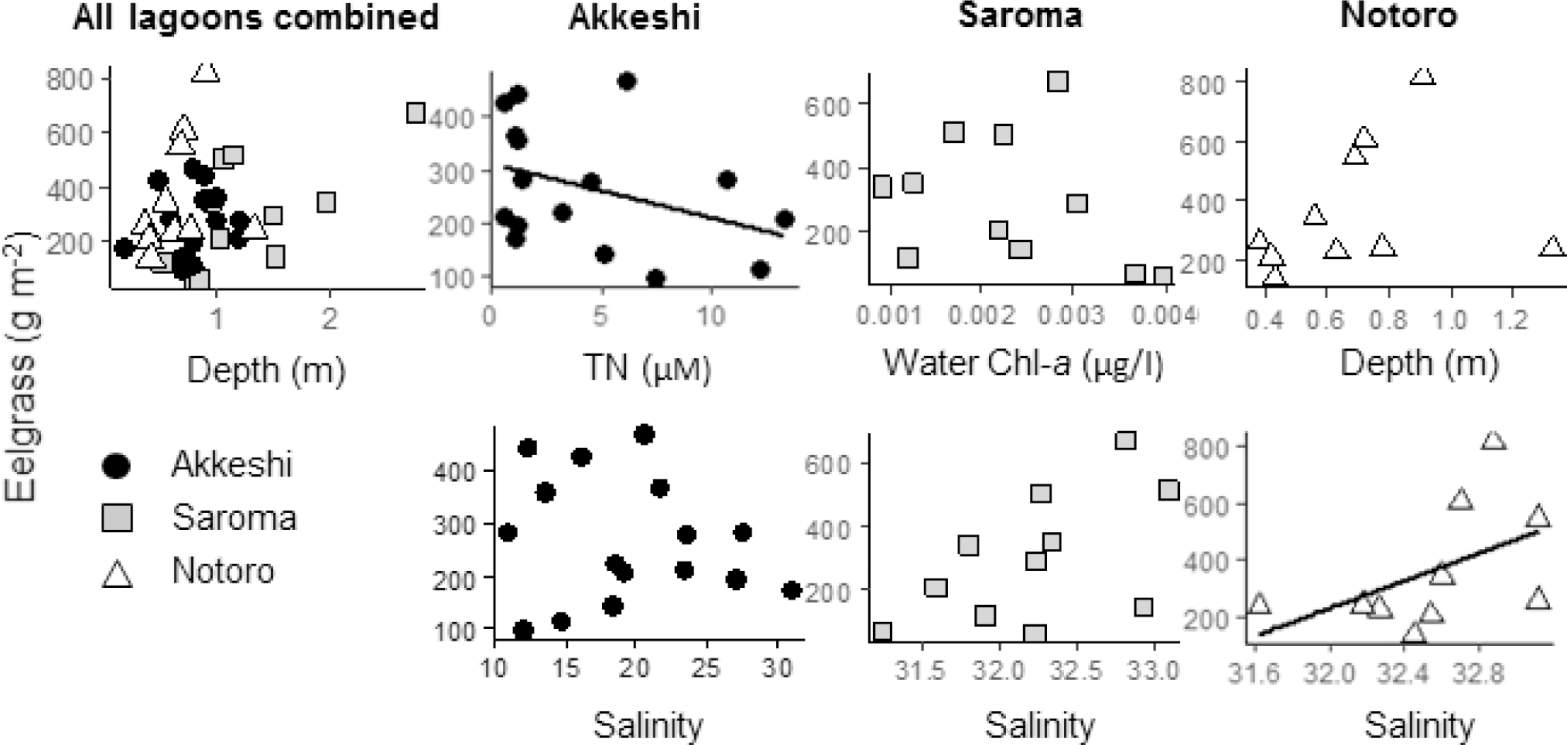
Scatterplots showing the relationships between eelgrass (above-ground dry weight, g m^-2^) and the explanatory variables. The line was obtained from linear regression (only shown if the relationship between the two variables was significant; linear model, *P* ≤ 0.05), and the values for the explanatory variables (*P* ≤ 0.05) were chosen in the best LMM for all lagoons combined, Akkeshi, Saroma, and Notoro.

For the eelgrass biomass and microalgal abundance, the combinations of explanatory environmental factors differed in the best models (Table 4). Eelgrass was best described by depth (AICc 390.3) when all data from the three lagoons were combined, indicating that the biomass of eelgrass increases with water depth (Fig 4). The fixed factor explained 9% of the total variance of the model, and 54% of the variance was explained when the fixed factor was combined with the random factors (lagoon, site, and season). When the model was analyzed for each lagoon separately, the combinations of the fixed factors differed among lagoons. TN and salinity best described the eelgrass biomass in Akkeshi (AICc 156.5), the water Chl-*a* concentration and salinity best described the eelgrass biomass in Saroma (AICc 133.7), and depth and salinity best described the eelgrass biomass in Notoro (AICc 59.3).

**Table 4:** Results of the LMMs for the best combination of the environmental factors responsible for the variation in each producer group (eelgrass, microalgae, and epifauna).

	Response											
	Eelgrass				Microalgae				Epifauna			
	All	Akkeshi	Saroma	Notoro	All	Akkeshi	Saroma	Notoro	All	Akkeshi	Saroma	Notoro
**T values for Fixed Factors**												
(Intercepts)	**13.9**	**8.2**	**-0.7**	**-8.4**	**-1.7**	**4.6**	-2.1	-1.5	**-1.2**	0.4	**-2.8**	**-3.2**
TN		**-4.8**									**-3.9**	
Depth	**4.8**			**8.1**			**5.3**					
Water tempeature					**3.2**			1.4			**2.8**	
Salinity		**-3.7**	**0.6**	**8.8**								
Water Chl-a			**-4.1**			**3.6**	**-3.7**					
Eelgrass					**6.7**	**5.1**		**3.1**	**3.8**			**5.7**
Microalgae										1.3		
**Random Factors**												
**(Variance ± SD)**												
Site:Lagoon	0.0 ± 0.2				0.4 ± 0.6				0.2 ± 0.5			
Lagoon	0.1 ± 0.4				1.7 ± 1.3				0.4 ± 0.5			
Site		0.3 ± 0.5	0.3 ± 0.5	0.0 ± 0.0		0.8 ± 0.9	0.0 ± 0.0	0.2 ± 0.5		0.2 ± 0.4	0.3 ± 0.5	0.0 ± 0.0
Season	0.2 ± 0.5	0.0 ± 0.0	0.5 ± 0.7	0.0 ± 0.0	0.0 ± 0.0	0.0 ± 0.2	0.0 ± 0.0	0.0 ± 0.0	0.0 ± 0.0	0.0 ± 0.0	3.4 ± 1.8	0.1 ± 0.3
Residual	0.4 ± 0.6	0.2 ± 0.5	0.4 ± 0.6	0.0 ± 0.0	0.5 ± 0.7	0.2 ± 0.5	0.5 ± 0.7	0.7 ± 0.8	0.4 ± 0.6	0.3 ± 0.6	0.5 ± 0.7	0.2 ± 0.4
**AICc**												
Null	397.3	159.8	148.9	93.8	506.0	181.4	160.9	162.8	452.1	171.3	155.6	92.4
Full	403.0	159.0	149.0	81.3	498.9	186.9	166.0	147.9	424.7	199.6	173.6	114.8
**Optimal**	**390.3**	**156.5**	**133.7**	**59.3**	**468.1**	**164.1**	**144.3**	**141.4**	**413.5**	**170.4**	**150.4**	**78.3**
**Marginal R**^**2**^	0.09	0.29	0.18	0.66	0.07	0.13	0.44	0.19	0.04	0.03	0.36	0.35
**Conditional R**^**2**^	0.54	0.65	0.74	0.69	0.81	0.80	0.44	0.41	0.57	0.38	0.92	0.61

The table shows the results for all lagoons combined (All) and for each lagoon (Akkeshi, Saroma, and Notoro), which were chosen based on AICc. T values with significant *P*-values (*α* ≤ 0.05) are in bold.

**Fig 5:**
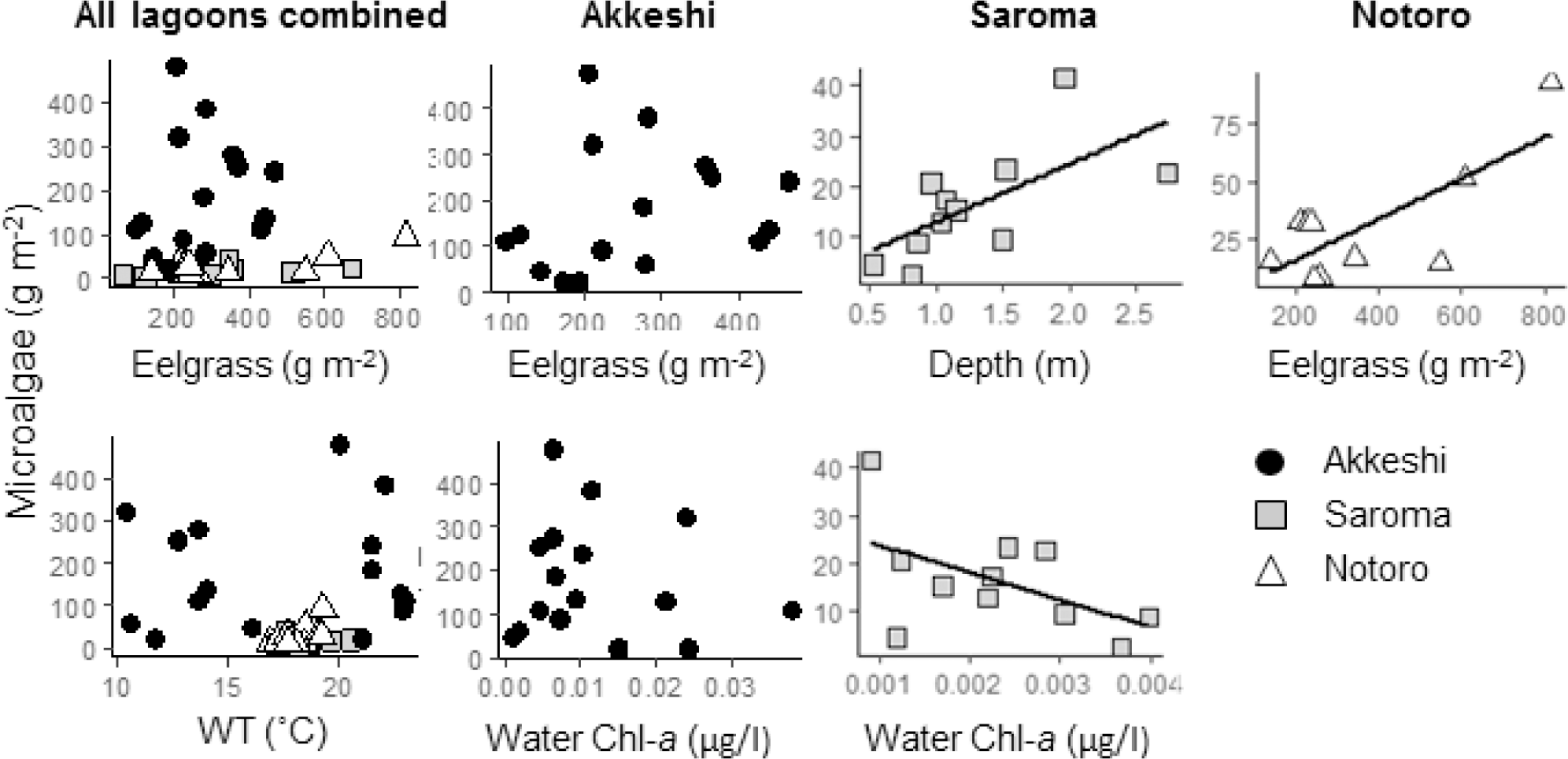
Scatterplots showing the relationships between microalgae weight (Chl*-a* g m^-2^) and the explanatory variables. The line was obtained from linear regression (only shown if the relationship between the two variables was significant; linear model, *P* ≤ 0.05), and the values for the explanatory variables (*P* ≤ 0.05) were chosen in the best LMM for all lagoons combined, Akkeshi, Saroma, and Notoro.

The microalgae abundance was best described by the combination of water temperature and eelgrass biomass (AICc 468.1), where the microalgae abundance increased with water temperature and eelgrass biomass (Fig 5). The two fixed factors together explained 7% of the total variance of the model, and 81% of the variance was explained by the fixed factors and three random factors. The best models analyzed for each of the three lagoons selected eelgrass biomass and water Chl-*a* for Akkeshi (AICc 164.1), depth and water Chl-*a* for Saroma (AICc 144.3), and eelgrass biomass and water temperature for Notoro (AICc 141.4).

**Fig 6:**
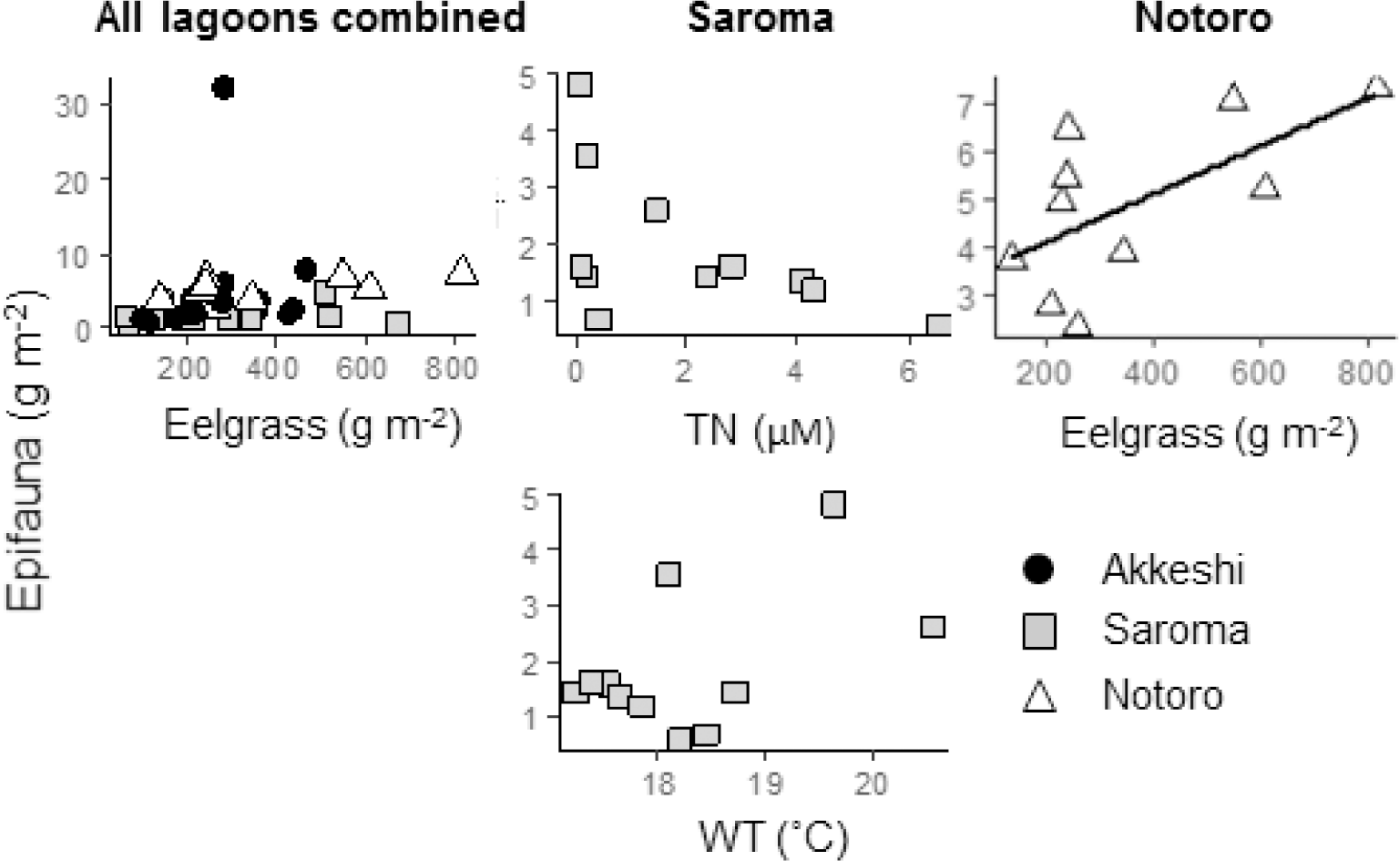
Scatterplots showing the relationships between epifauna (ash-free dry weight, g m^-2^) and the explanatory variables. The line was obtained from linear regression (only shown if the relationship between the two variables was significant; linear model, *P* ≤ 0.05), and the values for the explanatory variables (*P* ≤ 0.05) were chosen in the best LMM for all lagoons combined, Akkeshi, Saroma, and Notoro.

For epifauna, only the above-ground dry weight of eelgrass was selected as the explanatory variable of the best model (AICc 413.5; Table 4). This result showed that the epifaunal biomass increased with eelgrass biomass (Fig 6). The above-ground dry weight of eelgrass explained only 4% of the variance, whereas 57% of the total variance was explained by the three random factors and the fixed factor. Although not significant, the microalgae abundance was selected for the best model (AICc 170.4) for Akkeshi. TN and water temperature were selected for Saroma (AICc 150.4), and eelgrass biomass was selected for Notoro (AICc 78.3).

## Discussion

In this study, we assessed 1) the spatial scale at which eelgrass, epiphytic microalgae and epifauna vary the most, and 2) the abiotic/biotic factors that affect the observed spatial variability in producers and consumers in the eelgrass beds of eastern Hokkaido based on our hypotheses that that susceptibility of each functional group to these large and fine scale gradients is responsible for the spatial scale in which the differences in the biomass are the largest. We found that the amount of variation assigned to different spatial scales varied greatly among the different functional groups. For eelgrass and epifaunal biomass, the within-lagoon variation was greater than the among-lagoon variation. The factors that were responsible for the variation were related to fine scale environmental variations observed within each lagoon. In contrast, the differences in microalgae abundance were more prominent among lagoons than within lagoon. Furthermore, the combinations of responsible factors also varied among functional groups, suggesting that the processes causing the observed variations are different.

### Eelgrass

For eelgrass, the variables for explaining the variation in biomass are related to within-lagoon environmental gradients. Depth was selected as one of the predictor variables in the optimal models for each lagoon where eelgrass biomass was greater in deeper depths, which may be related to the taller canopy heights in deeper habitats. For example, SA5 was the deepest site in the Saroma-ko Lagoon, and the biomass was also highest at this site in the lagoon. For eelgrass, increased water depth limits light availability in the deeper parts of the bed, whereas eelgrass is subject to more intense stresses and disturbances in the shallower parts including the intertidal zone [68, 69]. Due to the constraints at both ends of the depth gradient, eelgrass biomass is generally maximized in the intermediate zone of its depth distribution [68, 70]. The observed pattern in this study indicated that the increase in eelgrass biomass occurred from the shallower limits to the optimum depth because our study sites were in the shallower areas of the subtidal zones where it is unlikely that light availability limits plant growth. Nevertheless, depth had overall a weak contribution in the model for all lagoons combined, and this indicates that it is not the most influential factor to explain the observed spatial variations.

Other responsible variables explaining the variation in eelgrass biomass were nutrient concentrations, the amount of phytoplankton in the water column (water Chl-*a*), and salinity, and these are also related to the environmental gradients within the lagoons. TN was a significant factor only in Akkeshi where nutrient-rich terrestrial water flows from the Bekanbeushi River [71]. In addition to the terrestrial sources, excess nutrients come from shellfish aquaculture [45], and the intensive oyster farming in Akkeshi may somewhat contribute to the presence of the within-lagoon nutrient gradients. The depletion of marine-originated nutrients at the time of eelgrass growth [37, 39] and the lack of both large river inflow and congregated aquacultural activities would explain why TN was not a significant factor in the two Okhotsk lagoons. The amount of water Chl-*a* is influenced by the water column nutrient concentration and can cause light attenuation in eelgrass bed ecosystems especially under eutrophic conditions [72]. This competition for the light resources between eelgrass and water column phytoplankton could be related to our observed pattern, and we could expand it to understand indirect negative effects of nutrients on eelgrass in relation to increase in water Chl-*a* for further research on the eelgrass bed ecosystem.

Salinity was selected as a predictor variable that was related to the variation in eelgrass biomass in each of the three lagoons. Similar to the observed nutrient input pattern, the salinity gradient within lagoon was most prominent in Akkeshi due to the presence of the large freshwater input volume from the Bekanbeushi River [24]. In contrast, Saroma and Notoro exhibited less obvious salinity gradients because of the strong and uniform tidal influences across the lagoons. Eelgrass can withstand a wide salinity range [73], but the levels of tolerance vary among populations [74]. More investigations are however required to understand the relationship between eelgrass biomass and salinity gradients.

### Microalgae

Unlike eelgrass, the results showed that the difference between the microalgae abundance in Akkeshi and the two sites along the Okhotsk coast (Saroma and Notoro) was great (up to 100-fold higher in Akkeshi), suggesting that the among-lagoon variation was greater than the within-lagoon variation. This difference might be related to the effects of the different ocean currents flowing along the two coasts. The water mass from the Oyashio cold current contained higher nutrient concentrations than that from the Soya warm current during the sampling seasons [36, 37, 39], which was consistent with our observations in the eelgrass beds. Also, the difference in water temperature is created by these two water currents, which is possibly related to the relationship between microalgae and water temperature observed from our results. It is possible that the higher nutrient concentrations along the Oyashio-influenced Pacific coast sustain the higher abundances, or biomass, of microalgae than along the Soya-influenced Okhotsk coast through various mechanisms such as supporting higher turnover rates [75]. Nevertheless, the effect of water temperature on microalgae remains unclear from this study and that the nutrient content was not selected as a predictor in our models, possibly because the nutrients from the ocean were already depleted at the time of sampling, and the rate of nutrient uptake by microalgae is faster than that by eelgrass [76].

Although microalgae biomass was best explained by the among-lagoon variation, there were still some effects of environmental variations observed within lagoons. Eelgrass biomass was one of the factors explaining the microalgal abundance in all lagoons except for Saroma. Aboveground eelgrass tissues provide substrata for the attachment of epiphytic microalgae [77], and eelgrass leaf emergence rate is also related to epiphytic algae load [78]. In addition, the negative relationship between the microalgal abundance and water Chl-*a* in two of the studied lagoons were observed. As both functional groups have similar light and nutrient requirements [79], the observed pattern may reflect the competitions for these resources among epiphytic microalgae and water column phytoplankton. In Akkeshi, on the other hand, a large portion of the Chl-*a* in the water comes from benthic microalgae that are detached and resuspended in the water column and not from phytoplankton [43], and this explain the slight difference in the trend.

### Epifauna

Our variation partitioning results suggest that scale-specific variation patterns of epifauna are similar to those of eelgrass, in which the spatial variation at the within-lagoon level was much more pronounced than that at the among-lagoon level. The LMM for all lagoons combined, as well as that for Notoro, showed a relationship between epifaunal biomass and eelgrass biomass, suggesting a bottom-up regulation of the abundance of the animals as recorded in previous studies [44, 80]. Gustafsson and Boström [81] reported that the increase in plant biomass that is followed by an increase in seagrass species richness is related to the increase in the associated faunal abundance. Leopardas et al. [82] presented the effect of aboveground seagrass biomass on epifaunal species composition. The ecosystem functions of an eelgrass bed include both habitat provisioning and the supply of food resources to the epifauna [1, 9, 83]. The epifaunal invertebrate grazers studied here consisted mostly of gammaridea, caprellidea, and gastropoda that directly consume epiphytic microalgae on eelgrass blades [84, 85]. For these species, it is likely that the eelgrass biomass provides important habitat space and foraging ground [86–88], which could explain the observed pattern regarding to eelgrass and epifaunal biomass and the similarity in the spatial variance pattern over multiple scales. As we see from the interaction between microalgae and eelgrass, and the fact that many epifauna feed on microalgae, there are possible implications for a trophic interaction in the studied eelgrass bed ecosystem. Nevertheless, our result does not show a clear link between microalgae biomass and epifauna biomass, and the observed pattern suggests that the plant-animal interactions in the studied eelgrass beds are more influenced by non-trophic interactions, such as habitat provisioning, than trophic interactions.

There were major differences in the taxonomic composition of epifauna within and among the lagoons, and among the temporal replicates. Although the results of the models for all lagoons combined did not show any effects of abiotic factors on the total epifaunal biomass, we found that TN and water temperature were affecting the epifauna in some degree, which may be explained by the differences in the compositions and functions of the epifaunal communities. For example, nutrient gradients caused by regional-scale eutrophication events [89] as well as regional differences in water temperature [90] are some of the known causes of variations in species composition.

## Conclusion

This research fulfills the needs for a local-scale assessment of the functions and services provided by coastal marine ecosystems by examining the spatial variation in the biomasses of different functional groups of plants and animals in eelgrass beds found within three lagoons with unique environmental properties. As expected, differences in the amount of freshwater input and terrestrial runoff from the river input to the lagoons and the current regimes create both among- and within-lagoon environmental variations. Moreover, different patterns of spatial-scale dependency in the variation in the different functional groups, and different combinations of predictors explained the patterns of variation in these organisms were observed from this study. Specifically, we found that those functional groups that exhibit more within- than among-lagoon variations in biomass are influenced by finer scale environmental gradients, and the group that has higher among-lagoon variation is more affected by large scale environmental factors. Thus, it is important to consider and analyze abiotic/biotic variables at multiple spatial scales when assessing the ecosystem functions and services of coastal and estuarine ecosystems, including seagrass beds. Also, future studies should take into account of the differences in environmental properties among various lagoons and other study areas as a same ecosystem can be affected by different combinations of environmental factors based on the properties of the surrounding habitats.

## Supporting information

S1 Dataset The environmental factors and biomass of eelgrass, microalgae, and epifauna. (XLSX)

S1 Text R commands used for the statistical analyses. (DOCX)

S1 Table Water column nutrients (μM) and water Chl-a concentrations (μg/l) in the summer and fall 2016 at the study sites in Akkeshi, Saroma, and Notoro. (DOCX)

S1 Fig Mean biomass of epifauna (g m^-2^; n = 5). The biomass is expressed by 9 major taxa for each site in Akkeshi, Saroma, and Notoro in the summer and fall. Minor taxonomical groups were grouped to one category (shown as Others). The symbols (*) on the graphs indicate no data.
(PDF)
