# Supplementary material for: Spatial patterns and predictor variables vary among different types of primary producers and consumers in eelgrass (Zostera marina) beds
Source: PLoS One. 2018 Aug 7;13(8):e0201791. doi: 10.1371/journal.pone.0201791 (PMC6080780; doi:10.1371/journal.pone.0201791)
Supplement: S1 Text — (DOCX) [file pone.0201791.s002.docx]

**S1 Text. R commands used for the statistical analyses.**

**Nested ANOVA and variation partitioning**

###### All lagoons combined ######

####DAGW (eelgrass dry above ground weight) as primary production####

#for SUMMER

library(lme4)

library(VCA)

setwd("C:/ ")

All<-read.csv("abundance_eelgrass.csv",h=T)

str(All)

All<-All[1:93,]

All$DAGW<-log(All$DAGW) #log transformation of size data

shapiro.test(All$DAGW)

bartlett.test(All$DAGW,All1$Lagoon,All1$Site)

ANOVA<-lm(DAGW~Lagoon/Site,data=All)

ANOVA

anova(ANOVA)

ANOVA<-aov(DAGW~Lagoon/Site,data=All) # for post hoc

ph<-TukeyHSD(x=ANOVA,'Lagoon',conf.level=0.95) # for post hoc

ph

m1<-anovaVCA(DAGW~Lagoon+Lagoon/Site,All)

inf<-VCAinference(m1,VarVC = TRUE)

print(inf,what="VCA")

par(mfrow = c(2, 2)) #testing for the assumptions

plot(m1)

#FALL

All<-read.csv("abundance_eelgrass.csv",h=T)

All1<-All[94:183,]

All1$DAGW<-log(All1$DAGW) #log transformation of size data

shapiro.test(All1$DAGW)

bartlett.test(All1$DAGW,All1$Lagoon,All1$Site)

ANOVA2<-lm(DAGW~Lagoon/Site,data=All1)

anova(ANOVA2)

ANOVA<-aov(DAGW~Lagoon/Site,data=All) # for post hoc

ph<-TukeyHSD(x=ANOVA,'Lagoon',conf.level=0.95) # for post hoc

ph

m2<-anovaVCA(DAGW~Lagoon+Lagoon/Site,All1)

inf<-VCAinference(m2,VarVC = TRUE)

print(inf,what="VCA")

par(mfrow = c(2, 2)) #testing for the assumptions

plot(m2)

####epiphytic Epip_Chla biomass as primary production####

#SUMMER

All2<-read.csv("abundance_Epip_Chla.csv")

str(All2)

All2<-All2[1:93,]

All2$Epip_Chla<-log(All2$Epip_Chla) #log transformation of size data

shapiro.test(All2$Epip_Chla)

bartlett.test(All2$Epip_Chla,All2$Lagoon,All2$Site)

m2<-lm(Epip_Chla~Lagoon+Lagoon/Site, data=All2)

anova(m2)

ANOVA<-aov(Epip_Chla~Lagoon/Site,data=All2) # for post hoc

ph<-TukeyHSD(x=ANOVA,'Lagoon',conf.level=0.95) # for post hoc

ph

m2<-anovaVCA(Epip_Chla~Lagoon+Lagoon/Site,All2)

inf<-VCAinference(m2,VarVC = TRUE)

print(inf,what="VCA")

#FALL

All2<-read.csv("abundance_Epip_Chla.csv")

All2<-All2[94:182,]

All2$Epip_Chla<-log(All2$Epip_Chla) #log transformation of size data

shapiro.test(All2$Epip_Chla)

bartlett.test(All2$Epip_Chla,All2$Lagoon,All2$Site)

m2<-lm(Epip_Chla~Lagoon+Lagoon/Site, data=All2)

anova(m2)

ANOVA<-aov(Epip_Chla~Lagoon/Site,data=All2) # for post hoc

ph<-TukeyHSD(x=ANOVA,'Lagoon',conf.level=0.95) # for post hoc

ph

m2<-anovaVCA(Epip_Chla~Lagoon+Lagoon/Site,All2)

inf<-VCAinference(m2,VarVC = TRUE)

print(inf,what="VCA")

####epifauna ash-free dry weight####

#SUMMER

Epi<-read.csv("abundance_epifauna.csv",h=T)

str(Epi)

Epi<-Epi[1:95,]

Epi$Epi<-log(Epi$Epi) #sqrt transformation of size data

shapiro.test(Epi$Epi)

bartlett.test(Epi$Epi,Epi$Lagoon,Epi$Site)

m2<-lm(Epi~Lagoon+Lagoon/Site, data=Epi)

anova(m2)

ANOVA<-aov(Epi~Lagoon/Site,data=Epi) # for post hoc

ph<-TukeyHSD(x=ANOVA,'Lagoon',conf.level=0.95) # for post hoc

ph

m2<-anovaVCA(Epi~Lagoon+Lagoon/Site,Epi)

inf<-VCAinference(m2,VarVC = TRUE)

print(inf,what="VCA")

#FALL

Epi<-read.csv("abundance_epifauna.csv",h=T)

str(Epi)

Epi<-Epi[96:185,]

Epi$Epi<-log(Epi$Epi) #log transformation of size data

shapiro.test(Epi$Epi)

bartlett.test(Epi$Epi,Epi$Lagoon,Epi$Site)

m2<-lm(Epi~Lagoon+Lagoon/Site, data=Epi)

anova(m2)

ANOVA<-aov(Epi~Lagoon/Site,data=Epi) # for post hoc

ph<-TukeyHSD(x=ANOVA,'Lagoon',conf.level=0.95) # for post hoc

ph

m2<-anovaVCA(Epi~Lagoon+Lagoon/Site,Epi)

inf<-VCAinference(m2,VarVC = TRUE)

print(inf,what="VCA")

**Linear mixed model with AICc selection and *R^2^* value calculation**

library(MuMIn)

library(nlme)

library(bbmle)

library(glmmML)

library(lmerTest)

library(Hmisc)

library(GGally)

library(MASS)

setwd("C:/ ")

LMM<-read.csv("Abiotic_biotic.csv",h=T)

str(LMM)

LMM[,6:17]<-log(LMM[,6:17]) #log transformation

str(LMM)

par(mfrow = c(2, 2)) #testing for the assumptions

cor(LMM[,6:17], use="complete.obs", method="pearson") # for checking Pearsons correlation

rcorr(as.matrix(LMM[,6:17]))

ggpairs(LMM[,c("Epip_Chla","Water_Chla","DAGW","Epi", "SWT","PO4.P","TN","Depth","WT","Salinity")])

##the rcorr result shows that SWT-WT and PO4-TN are causing multicollinearity so we get rid of PO4 and SWT

shapiro.test(LMM$DAGW)

shapiro.test(LMM$Water_Chla)

shapiro.test(LMM$Epi)

shapiro.test(LMM$SWT)

shapiro.test(LMM$TN)

shapiro.test(LMM$WT)

shapiro.test(LMM$Salinity)

shapiro.test(LMM$Depth)

#######Eelgrass

A<-LMM[c(31:70,121:160),]

E<-lmer(DAGW~TN+Depth+WT+Salinity+Water_Chla+(1|Lagoon/Site)+(1|Season),data=LMM,REML = TRUE)

E

m1.1<-lmer(DAGW~TN+(1|Lagoon/Site)+(1|Season),data=LMM,REML = TRUE)

m1.2<-lmer(DAGW~Depth+(1|Lagoon/Site)+(1|Season),data=LMM,REML = TRUE)

m1.3<-lmer(DAGW~WT+(1|Lagoon/Site)+(1|Season),data=LMM,REML = TRUE)

m1.4<-lmer(DAGW~Salinity+(1|Lagoon/Site)+(1|Season),data=LMM,REML = TRUE)

m1.5<-lmer(DAGW~Water_Chla+(1|Lagoon/Site)+(1|Season),data=LMM,REML = TRUE)

m1.6<-lmer(DAGW~TN+Depth+(1|Lagoon/Site)+(1|Season),data=LMM,REML = TRUE)

m1.7<-lmer(DAGW~TN+WT+(1|Lagoon/Site)+(1|Season),data=LMM,REML = TRUE)

m1.8<-lmer(DAGW~TN+Salinity+(1|Lagoon/Site)+(1|Season),data=LMM,REML = TRUE)

m1.9<-lmer(DAGW~TN+Water_Chla+(1|Lagoon/Site)+(1|Season),data=LMM,REML = TRUE)

m1.10<-lmer(DAGW~Depth+WT+(1|Lagoon/Site)+(1|Season),data=LMM,REML = TRUE)

m1.11<-lmer(DAGW~Depth+Salinity+(1|Lagoon/Site)+(1|Season),data=LMM,REML = TRUE)

m1.12<-lmer(DAGW~Depth+Water_Chla+(1|Lagoon/Site)+(1|Season),data=LMM,REML = TRUE)

m1.13<-lmer(DAGW~WT+Salinity+(1|Lagoon/Site)+(1|Season),data=LMM,REML = TRUE)

m1.14<-lmer(DAGW~WT+Water_Chla+(1|Lagoon/Site)+(1|Season),data=LMM,REML = TRUE)

m1.15<-lmer(DAGW~Salinity+Water_Chla+(1|Lagoon/Site)+(1|Season),data=LMM,REML = TRUE)

m1.16<-lmer(DAGW~TN+Depth+WT+(1|Lagoon/Site)+(1|Season),data=LMM,REML = TRUE)

m1.17<-lmer(DAGW~TN+Depth+Salinity+(1|Lagoon/Site)+(1|Season),data=LMM,REML = TRUE)

m1.18<-lmer(DAGW~TN+Depth+Water_Chla+(1|Lagoon/Site)+(1|Season),data=LMM,REML = TRUE)

m1.19<-lmer(DAGW~Depth+WT+Salinity+(1|Lagoon/Site)+(1|Season),data=LMM,REML = TRUE)

m1.20<-lmer(DAGW~Depth+WT+Water_Chla+(1|Lagoon/Site)+(1|Season),data=LMM,REML = TRUE)

m1.21<-lmer(DAGW~WT+Salinity+Water_Chla+(1|Lagoon/Site)+(1|Season),data=LMM,REML = TRUE)

m1.22<-lmer(DAGW~TN+Depth+WT+Salinity+(1|Lagoon/Site)+(1|Season),data=LMM,REML = TRUE)

m1.23<-lmer(DAGW~TN+Depth+WT+Water_Chla+(1|Lagoon/Site)+(1|Season),data=LMM,REML = TRUE)

m1.24<-lmer(DAGW~TN+Depth+Salinity+Water_Chla+(1|Lagoon/Site)+(1|Season),data=LMM,REML = TRUE)

m1.25<-lmer(DAGW~TN+Depth+WT+Salinity+Water_Chla+(1|Lagoon/Site)+(1|Season),data=LMM,REML = TRUE)

m1.26<-lmer(DAGW~1+(1|Lagoon/Site)+(1|Season),data=LMM,REML = TRUE)

AICctab(E,m1.1,m1.2,m1.3,m1.4,m1.5,m1.6,m1.7,m1.8,m1.9,m1.10,m1.11,m1.12,m1.13,m1.14,m1.15,m1.16,m1.17,m1.18,m1.19,m1.20,m1.21,m1.22,m1.23,m1.24,m1.26,base=T,weights=T,nobs=length(LMM))

#m1.2 as the best model

summary(m1.2)

anova(m1.2)

r.squaredGLMM(m1.2)

#Microalgae

E<-lmer(Epip_Chla~TN+Depth+WT+Water_Chla+DAGW+(1|Lagoon/Site)+(1|Season),data=LMM,REML = TRUE)

m1.1<-lmer(Epip_Chla~TN+(1|Lagoon/Site)+(1|Season),data=LMM,REML = TRUE)

m1.2<-lmer(Epip_Chla~DAGW+(1|Lagoon/Site)+(1|Season),data=LMM,REML = TRUE)

m1.3<-lmer(Epip_Chla~Depth+(1|Lagoon/Site)+(1|Season),data=LMM,REML = TRUE)

m1.4<-lmer(Epip_Chla~WT+(1|Lagoon/Site)+(1|Season),data=LMM,REML = TRUE)

m1.5<-lmer(Epip_Chla~Water_Chla+(1|Lagoon/Site)+(1|Season),data=LMM,REML = TRUE)

m1.6<-lmer(Epip_Chla~TN+DAGW+(1|Lagoon/Site)+(1|Season),data=LMM,REML = TRUE)

m1.7<-lmer(Epip_Chla~TN+Depth+(1|Lagoon/Site)+(1|Season),data=LMM,REML = TRUE)

m1.8<-lmer(Epip_Chla~TN+WT+(1|Lagoon/Site)+(1|Season),data=LMM,REML = TRUE)

m1.9<-lmer(Epip_Chla~TN+Water_Chla+(1|Lagoon/Site)+(1|Season),data=LMM,REML = TRUE)

m1.10<-lmer(Epip_Chla~DAGW+Depth+(1|Lagoon/Site)+(1|Season),data=LMM,REML = TRUE)

m1.11<-lmer(Epip_Chla~DAGW+WT+(1|Lagoon/Site)+(1|Season),data=LMM,REML = TRUE)

m1.12<-lmer(Epip_Chla~DAGW+Water_Chla+(1|Lagoon/Site)+(1|Season),data=LMM,REML = TRUE)

m1.13<-lmer(Epip_Chla~Depth+WT+(1|Lagoon/Site)+(1|Season),data=LMM,REML = TRUE)

m1.14<-lmer(Epip_Chla~Depth+Water_Chla+(1|Lagoon/Site)+(1|Season),data=LMM,REML = TRUE)

m1.15<-lmer(Epip_Chla~WT+Water_Chla+(1|Lagoon/Site)+(1|Season),data=LMM,REML = TRUE)

m1.16<-lmer(Epip_Chla~TN+DAGW+Depth+(1|Lagoon/Site)+(1|Season),data=LMM,REML = TRUE)

m1.17<-lmer(Epip_Chla~TN+DAGW+WT+(1|Lagoon/Site)+(1|Season),data=LMM,REML = TRUE)

m1.18<-lmer(Epip_Chla~TN+DAGW+Water_Chla+(1|Lagoon/Site)+(1|Season),data=LMM,REML = TRUE)

m1.19<-lmer(Epip_Chla~DAGW+Depth+WT+(1|Lagoon/Site)+(1|Season),data=LMM,REML = TRUE)

m1.20<-lmer(Epip_Chla~DAGW+Depth+Water_Chla+(1|Lagoon/Site)+(1|Season),data=LMM,REML = TRUE)

m1.21<-lmer(Epip_Chla~Depth+WT+Water_Chla+(1|Lagoon/Site)+(1|Season),data=LMM,REML = TRUE)

m1.22<-lmer(Epip_Chla~TN+DAGW+Depth+WT+(1|Lagoon/Site)+(1|Season),data=LMM,REML = TRUE)

m1.23<-lmer(Epip_Chla~TN+DAGW+Depth+Water_Chla+(1|Lagoon/Site)+(1|Season),data=LMM,REML = TRUE)

m1.24<-lmer(Epip_Chla~TN+DAGW+WT+Water_Chla+(1|Lagoon/Site)+(1|Season),data=LMM,REML = TRUE)

m1.25<-lmer(Epip_Chla~TN+DAGW+WT+Water_Chla+(1|Lagoon/Site)+(1|Season),data=LMM,REML = TRUE)

m1.26<-lmer(Epip_Chla~TN+DAGW+Depth+WT+Water_Chla+(1|Lagoon/Site)+(1|Season),data=LMM,REML = TRUE)

m1.27<-lmer(Epip_Chla~1+(1|Lagoon/Site)+(1|Season),data=LMM,REML = TRUE)

AICctab(E,m1.1,m1.2,m1.3,m1.4,m1.5,m1.6,m1.7,m1.8,m1.9,m1.10,m1.11,m1.12,m1.13,m1.14,m1.15,m1.16,m1.17,m1.18,m1.19,m1.20,m1.21,m1.22,m1.23,m1.24,m1.26,m1.27,base=T,weights=T,nobs=length(LMM))

#m1.11 as best model

summary(m1.11)

anova(m1.11)

r.squaredGLMM(m1.11)

#epifauna

m3<-lmer(Epi~Epip_Chla+Water_Chla+TN+Depth+WT+(1|Lagoon/Site)+(1|Season),data=LMM,REML=TRUE) #for DAGW

m3.1<-lmer(Epi~Epip_Chla+(1|Lagoon/Site)+(1|Season),data=LMM,REML=TRUE)

m3.2<-lmer(Epi~Water_Chla+(1|Lagoon/Site)+(1|Season),data=LMM,REML=TRUE)

m3.3<-lmer(Epi~DAGW+(1|Lagoon/Site)+(1|Season),data=LMM,REML=TRUE)

m3.4<-lmer(Epi~TN+(1|Lagoon/Site)+(1|Season),data=LMM,REML=TRUE)

m3.5<-lmer(Epi~Depth+(1|Lagoon/Site)+(1|Season),data=LMM,REML=TRUE)

m3.6<-lmer(Epi~WT+(1|Lagoon/Site)+(1|Season),data=LMM,REML=TRUE)

m3.7<-lmer(Epi~Epip_Chla+Water_Chla+(1|Lagoon/Site)+(1|Season),data=LMM,REML=TRUE)

m3.8<-lmer(Epi~Epip_Chla+DAGW+(1|Lagoon/Site)+(1|Season),data=LMM,REML=TRUE)

m3.9<-lmer(Epi~Epip_Chla+TN+(1|Lagoon/Site)+(1|Season),data=LMM,REML=TRUE)

m3.10<-lmer(Epi~Epip_Chla+Depth+(1|Lagoon/Site)+(1|Season),data=LMM,REML=TRUE)

m3.11<-lmer(Epi~Epip_Chla+WT+(1|Lagoon/Site)+(1|Season),data=LMM,REML=TRUE)

m3.12<-lmer(Epi~Water_Chla+DAGW+(1|Lagoon/Site)+(1|Season),data=LMM,REML=TRUE)

m3.13<-lmer(Epi~Water_Chla+TN+(1|Lagoon/Site)+(1|Season),data=LMM,REML=TRUE)

m3.14<-lmer(Epi~Water_Chla+Depth+(1|Lagoon/Site)+(1|Season),data=LMM,REML=TRUE)

m3.15<-lmer(Epi~Water_Chla+WT+(1|Lagoon/Site)+(1|Season),data=LMM,REML=TRUE)

m3.16<-lmer(Epi~DAGW+TN+(1|Lagoon/Site)+(1|Season),data=LMM,REML=TRUE)

m3.17<-lmer(Epi~DAGW+Depth+(1|Lagoon/Site)+(1|Season),data=LMM,REML=TRUE)

m3.18<-lmer(Epi~DAGW+WT+(1|Lagoon/Site)+(1|Season),data=LMM,REML=TRUE)

m3.19<-lmer(Epi~TN+Depth+(1|Lagoon/Site)+(1|Season),data=LMM,REML=TRUE)

m3.20<-lmer(Epi~TN+WT+(1|Lagoon/Site)+(1|Season),data=LMM,REML=TRUE)

m3.21<-lmer(Epi~Depth+WT+(1|Lagoon/Site)+(1|Season),data=LMM,REML=TRUE)

m3.22<-lmer(Epi~Epip_Chla+Water_Chla+DAGW+(1|Lagoon/Site)+(1|Season),data=LMM,REML=TRUE)

m3.23<-lmer(Epi~Epip_Chla+Water_Chla+TN+(1|Lagoon/Site)+(1|Season),data=LMM,REML=TRUE)

m3.24<-lmer(Epi~Epip_Chla+Water_Chla+Depth+(1|Lagoon/Site)+(1|Season),data=LMM,REML=TRUE)

m3.25<-lmer(Epi~Epip_Chla+Water_Chla+WT+(1|Lagoon/Site)+(1|Season),data=LMM,REML=TRUE)

m3.26<-lmer(Epi~Epip_Chla+DAGW+TN+(1|Lagoon/Site)+(1|Season),data=LMM,REML=TRUE)

m3.27<-lmer(Epi~Epip_Chla+DAGW+Depth+(1|Lagoon/Site)+(1|Season),data=LMM,REML=TRUE)

m3.28<-lmer(Epi~Epip_Chla+DAGW+WT+(1|Lagoon/Site)+(1|Season),data=LMM,REML=TRUE)

m3.29<-lmer(Epi~Epip_Chla+TN+WT+(1|Lagoon/Site)+(1|Season),data=LMM,REML=TRUE)

m3.30<-lmer(Epi~TN+Water_Chla+DAGW+(1|Lagoon/Site)+(1|Season),data=LMM,REML=TRUE)

m3.31<-lmer(Epi~TN+Water_Chla+Depth+(1|Lagoon/Site)+(1|Season),data=LMM,REML=TRUE)

m3.32<-lmer(Epi~TN+Water_Chla+WT+(1|Lagoon/Site)+(1|Season),data=LMM,REML=TRUE)

m3.33<-lmer(Epi~TN+DAGW+Depth+(1|Lagoon/Site)+(1|Season),data=LMM,REML=TRUE)

m3.34<-lmer(Epi~TN+DAGW+WT+(1|Lagoon/Site)+(1|Season),data=LMM,REML=TRUE)

m3.35<-lmer(Epi~TN+Depth+WT+(1|Lagoon/Site)+(1|Season),data=LMM,REML=TRUE)

m3.36<-lmer(Epi~Water_Chla+DAGW+Depth+(1|Lagoon/Site)+(1|Season),data=LMM,REML=TRUE)

m3.37<-lmer(Epi~Water_Chla+DAGW+WT+(1|Lagoon/Site)+(1|Season),data=LMM,REML=TRUE)

m3.38<-lmer(Epi~Water_Chla+Depth+WT+(1|Lagoon/Site)+(1|Season),data=LMM,REML=TRUE)

m3.39<-lmer(Epi~DAGW+Depth+WT+(1|Lagoon/Site)+(1|Season),data=LMM,REML=TRUE)

m3.40<-lmer(Epi~Epip_Chla+TN+Water_Chla+DAGW+(1|Lagoon/Site)+(1|Season),data=LMM,REML=TRUE)

m3.41<-lmer(Epi~Epip_Chla+TN+Water_Chla+Depth+(1|Lagoon/Site)+(1|Season),data=LMM,REML=TRUE)

m3.42<-lmer(Epi~Epip_Chla+TN+Water_Chla+WT+(1|Lagoon/Site)+(1|Season),data=LMM,REML=TRUE)

m3.43<-lmer(Epi~Epip_Chla+TN+DAGW+Depth+(1|Lagoon/Site)+(1|Season),data=LMM,REML=TRUE)

m3.44<-lmer(Epi~Epip_Chla+TN+DAGW+WT+(1|Lagoon/Site)+(1|Season),data=LMM,REML=TRUE)

m3.45<-lmer(Epi~Epip_Chla+TN+Depth+WT+(1|Lagoon/Site)+(1|Season),data=LMM,REML=TRUE)

m3.46<-lmer(Epi~TN+Water_Chla+DAGW+Depth+(1|Lagoon/Site)+(1|Season),data=LMM,REML=TRUE)

m3.47<-lmer(Epi~TN+Water_Chla+DAGW+WT+(1|Lagoon/Site)+(1|Season),data=LMM,REML=TRUE)

m3.48<-lmer(Epi~TN+DAGW+Depth+WT+(1|Lagoon/Site)+(1|Season),data=LMM,REML=TRUE)

m3.49<-lmer(Epi~Water_Chla+DAGW+Depth+WT+(1|Lagoon/Site)+(1|Season),data=LMM,REML=TRUE)

m3.50<-lmer(Epi~Epip_Chla+TN+Water_Chla+DAGW+Depth+(1|Lagoon/Site)+(1|Season),data=LMM,REML=TRUE)

m3.51<-lmer(Epi~Epip_Chla+TN+Water_Chla+DAGW+WT+(1|Lagoon/Site)+(1|Season),data=LMM,REML=TRUE)

m3.52<-lmer(Epi~Epip_Chla+TN+DAGW+Depth+WT+(1|Lagoon/Site)+(1|Season),data=LMM,REML=TRUE)

m3.53<-lmer(Epi~Epip_Chla+Water_Chla+DAGW+Depth+WT+(1|Lagoon/Site)+(1|Season),data=LMM,REML=TRUE)

m3.54<-lmer(Epi~TN+Water_Chla+DAGW+Depth++WT+(1|Lagoon/Site)+(1|Season),data=LMM,REML=TRUE)

m3.55<-lmer(Epi~Epip_Chla+TN+Water_Chla+DAGW+Depth+WT+(1|Lagoon/Site)+(1|Season),data=LMM,REML=TRUE)

m3.56<-lmer(Epi~1+(1|Lagoon/Site)+(1|Season),data=LMM,REML=TRUE)

AICctab(m3,m3.1,m3.2,m3.3,m3.4,m3.5,m3.6,m3.7,m3.8,m3.9,m3.10,m3.11,m3.12,m3.13,m3.14,m3.15,m3.16,

m3.17,m3.18,m3.19,m3.20,m3.21,m3.22,m3.23,m3.24,m3.25,m3.26,m3.27,m3.28,m3.29,m3.30,m3.31,m3.32,m3.33,m3.34,m3.35,m3.36,m3.37,m3.38,m3.39,m3.40,m3.41,

m3.42,m3.43,m3.44,m3.45,m3.46,m3.47,m3.48,m3.49,m3.50,m3.51,m3.52,m3.53,m3.54,m3.55,m3.56,base=T,weights=T,nobs=length(LMM))

#m3.3 as the best model

m3.3<-lmer(Epi~DAGW+(1|Lagoon/Site)+(1|Season),data=LMM,REML=TRUE)

summary(m3.3)

anova(m3.3)

r.squaredGLMM(m3.3)

##for each lagoon, repeat the analysis above ##
