# Supplementary material for: Spatial patterns and predictor variables vary among different types of primary producers and consumers in eelgrass (Zostera marina) beds
Source: PLoS One. 2018 Aug 7;13(8):e0201791. doi: 10.1371/journal.pone.0201791 (PMC6080780; doi:10.1371/journal.pone.0201791)
Supplement: S1 Table — (DOCX) [file pone.0201791.s003.docx]

**S1 Table. Water column nutrients (μM) and water Chl-a concentrations (μg/l) in the summer and fall 2016 at the study sites in Akkeshi, Saroma, and Notoro.**

| Site | PO_4_ | | NO_2_ | | NO_3_ | | NH_4_ | | Water Chl-*a* | |
| --- | --- | --- | --- | --- | --- | --- | --- | --- | --- | --- |
|  | Summer | Fall | Summer | Fall | Summer | Fall | Summer | Fall | Summer | Fall |
| **Akkeshi** |  |  |  |  |  |  |  |  |  |  |
| AK | 0.4 | 0.3 | 0.1 | 0.4 | 0.04 | 0.3 | 1.0 | 0.6 | 24.3 | 15.1 |
| BK | 0.3 | 3.2 | 0.3 | 0.9 | 4.1 | 3.6 | 0.7 | 6.3 | 1.0 | 11.5 |
| CK | 0.6 | 4.3 | 0.4 | 0.6 | 0.2 | 0.7 | 0.4 | 3.2 | 4.8 | 6.8 |
| CL | 0.3 | 5.0 | 0.4 | 0.7 | 0.5 | 0.5 | 0.5 | 4.9 | 1.9 | 10.2 |
| HN | 1.6 | 4.6 | 0.4 | 0.9 | 0.2 | 0.6 | 0.6 | 5.9 | 9.3 | 38.0 |
| SL | 0.9 | 7.2 | 0.1 | 0.6 | 0.002 | 0.3 | 0.5 | 2.3 | 4.6 | 7.2 |
| SR | 0.2 | 1.7 | 0.1 | 1.3 | 0.1 | 6.4 | 0.4 | 5.6 | 23.9 | 6.3 |
| TB | 1.3 | 6.7 | 0.5 | 1.0 | 0.2 | 1.9 | 0.5 | 9.3 | 6.4 | 21.2 |
| Mean±SD | 0.7 ± 0.5 | 4.1 ± 2.4 | 0.3 ± 0.2 | 0.8 ± 0.3 | 0.7 ± 1.4 | 1.8 ± 2.2 | 0.6 ± 0.2 | 4.8 ± 2.7 | 9.5 ± 9.7 | 14.5 ± 10.7 |
| **Saroma** |  |  |  |  |  |  |  |  |  |  |
| SA1 | 0.1 | 1.4 | 0.04 | 0.4 | 0.02 | 1.4 | 0.2 | 2.4 | 3.0 | 4.9 |
| SA2 | n.d. | 1.4 | n.d. | 0.4 | n.d. | 1.6 | n.d. | 2.3 | n.d. | 8.1 |
| SA3 | 0.2 | 0.9 | 0.1 | 0.2 | 0.2 | 1.1 | 1.1 | 1.1 | 1.2 | 1.5 |
| SA5 | 0.08 | n.d. | 0.04 | n.d. | 0.01 | n.d. | 0.4 | n.d. | 2.8 | n.d. |
| SA6 | 0.08 | 0.7 | 0.02 | 0.2 | 0.005 | 2.2 | 0.06 | 4.1 | 2.2 | 3.3 |
| SA7 | 0.1 | n.d. | 0.03 | n.d. | 0.03 | n.d. | 0.2 | n.d. | 2.4 | n.d. |
| SA8 | 0.2 | 0.7 | 0.05 | 0.2 | 0.03 | 0.6 | 0.05 | 2.1 | 1.7 | 3.8 |
| Mean±SD | 0.1 ± 0.04 | 1.0 ± 0.4 | 0.05 ± 0.03 | 0.3 ± 0.1 | 0.05 ± 0.08 | 1.4 ± 0.6 | 0.3 ± 0.4 | 2.4 ± 1.1 | 2.2 ± 0.7 | 4.3 ± 2.5 |
| **Notoro** |  |  |  |  |  |  |  |  |  |  |
| NO1 | 0.3 | 0.9 | 0.02 | 0.1 | 0.03 | 0.4 | 0.4 | 0.2 | 2.6 | 9.0 |
| NO2 | 1.0 | 0.8 | 0.04 | 0.2 | 0.2 | 0.8 | 0.4 | 2.6 | 0.7 | 5.4 |
| NO3 | 0.1 | 0.4 | 0.04 | 0.2 | 0.07 | 0.5 | 0.8 | 0.9 | 1.0 | 2.3 |
| NO4 | 0.2 | 0.3 | 0.03 | 0.1 | 0.03 | 0.3 | 0.5 | 0.4 | 0.9 | 4.1 |
| NO5 | 0.4 | 0.8 | 0.1 | 0.09 | 0.4 | 0.4 | 0.66 | 0.2 | 0.6 | 2.6 |
| Mean±SD | 0.4 ± 0.3 | 0.6 ± 0.3 | 0.05 ± 0.03 | 0.1 ± 0.05 | 0.1 ± 0.1 | 0.5 ± 0.2 | 0.6 ± 0.2 | 0.8 ± 1.1 | 1.2 ± 0.8 | 4.7 ± 2.7 |

n.d. indicates no data available.
