# Supplementary material for: Spatial patterns and predictor variables vary among different types of primary producers and consumers in eelgrass (Zostera marina) beds
Source: PLoS One. 2018 Aug 7;13(8):e0201791. doi: 10.1371/journal.pone.0201791 (PMC6080780; doi:10.1371/journal.pone.0201791)

S1 Fig. Mean biomass of epifauna (g m^{-2}; n = 5). The biomass is expressed by 9 major taxa for each site in Akkeshi, Saroma, and Notoro in the summer and fall. Minor taxonomical groups were grouped to one category (shown as Others). The symbols (*) on the graphs indicate no data.

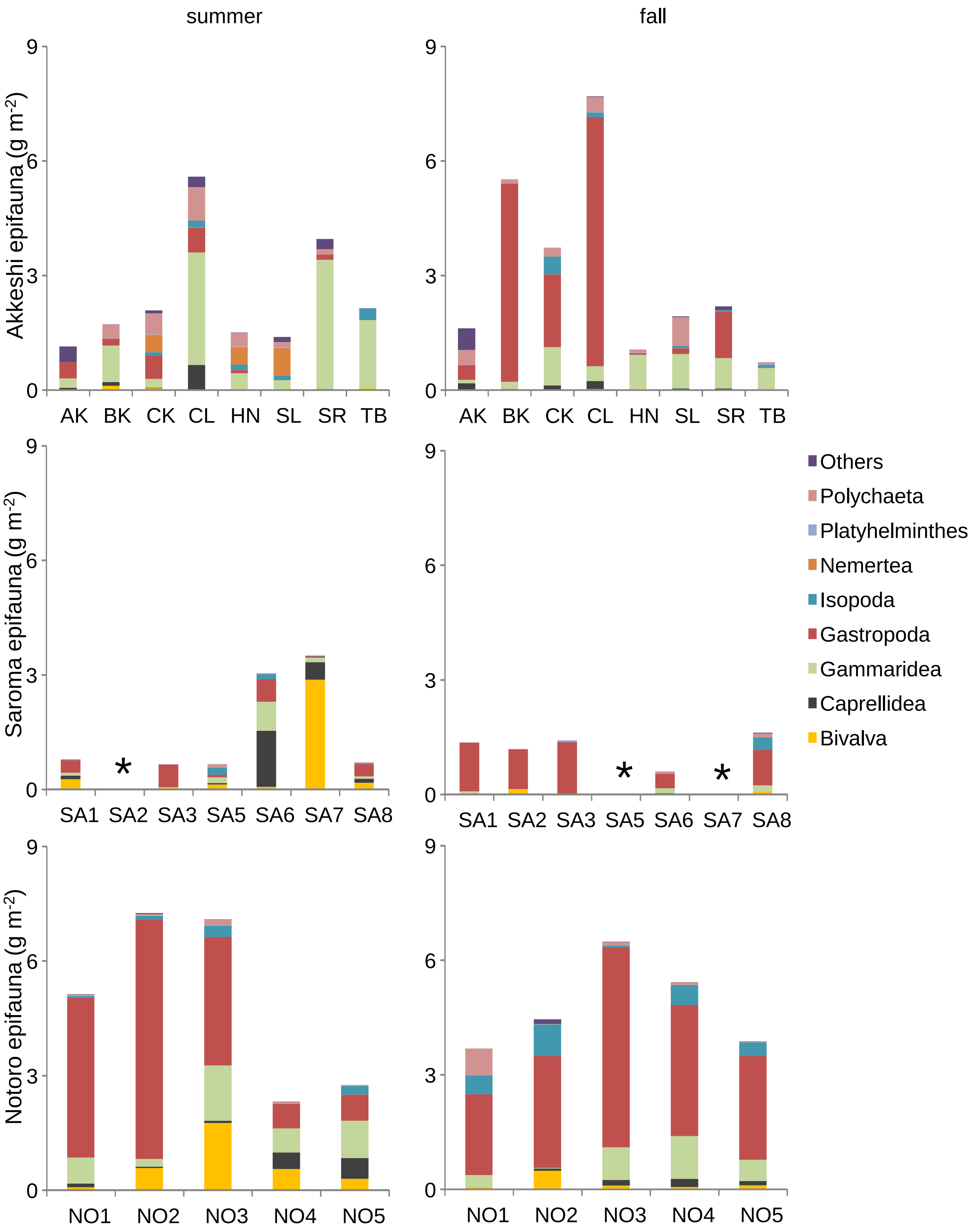

Supplement: S1 Fig — The biomass is expressed by 9 major taxa for each site in Akkeshi, Saroma, and Notoro in the summer and fall. Minor taxonomical groups were grouped to one category (shown as Others). The symbols (*) on the graphs indicate no data. (PDF) [file pone.0201791.s004.pdf]
